# Peri-Implantitis-Associated Microbiota before and after Peri-Implantitis Treatment, the Biofilm “Competitive Balancing” Effect: A Systematic Review of Randomized Controlled Trials

**DOI:** 10.3390/microorganisms12101965

**Published:** 2024-09-28

**Authors:** Federica Di Spirito, Massimo Pisano, Maria Pia Di Palo, Gianluigi Franci, Antonio Rupe, Antonino Fiorino, Carlo Rengo

**Affiliations:** 1 Department of Medicine, Surgery and Dentistry, University of Salerno, Via S. Allende, 84081 Baronissi, Italymariapia140497@gmail.com (M.P.D.P.); gfranci@unisa.it (G.F.); antoniorupe@virgilio.it (A.R.); 2 Department of Neuroscience, Reproductive Science and Dentistry, University of Naples Federico II, 80131 Naples, Italy; fiorinodr.antonino@gmail.com

**Keywords:** peri-implantitis, microbiome, microbiota, bacteria, dental implant, treatment

## Abstract

This systematic review of RCTs aimed to characterize short- and long-term changes in peri-implantitis-associated microbiota (total biofilm microbial load and predominant pathogens’ counts) following (any) peri-implantitis treatment in systemically healthy, non-smoking, partially/totally edentulous adults. The study protocol, compliant with the PRISMA statement, was registered on PROSPERO (CRD42024514521) before the literature search. Data from 11 RCTs, assessed through the ROBINS-2 tool, were qualitatively synthesized. No data were retrieved on total edentulism, healthy peri-implant/periodontal sites, treated mucositis, gingivitis, and periodontitis sites. Shortly after treatment, *Prevotella intermedia*, *Fusobacterium nucleatum*, and *Peptostreptococcus micros* prevailed, indicating early colonization, as after implant placement. After both surgical and non-surgical approaches, although not eradicated, the peri-implant total biofilm load, red- and orange-complex species, and *Aggregatibacter actinomycetemcomitans* counts generally decreased for up to about three months. However, one month after treatment, red-complex species and *Prevotella intermedia* increased, likely due to persistent tissue-invasive bacteria, unresolved pathological conditions (high probing depth values) favoring anaerobiosis and dysbiosis, and a qualitatively and quantitatively decreased biofilm community, competing and balancing the predominant pathogens (biofilm “competitive balancing” effect), thus allowing recolonization by more virulent bacteria. Red-complex bacteria gradually leveled off to baseline at the six- and twelve-month follow-ups. *Fusobacterium nucleatum* remained almost unchanged after treatment.

## 1. Introduction

Peri-implantitis is the most common late dental implant complication and affects approximately 15–57% of subjects and 8–28% of implants [1]. Its progression can lead to implant loss, which is estimated to occur in 8% of patients and 4% of implants [2].

Like periodontitis, peri-implantitis is an infectious inflammatory disease [3] sustained by the host’s inflammatory response triggered by oral dysbiosis [4,5,6]. In particular, the loss of balance in the oral microbiome, which is physiologically dependent on genetic and environmental factors such as diet, oral hygiene, stress, alcohol or smoking habits, pharmacological therapies (e.g., antibiotics and corticosteroids) [7], and various systemic or oral diseases [8,9,10,11], can lead to the onset of microbiota-associated oral diseases, such as peri-implantitis [8,9,12]. Dentition also influences oral and especially periodontal microbiota since dentate subjects have a more heterogeneous and richer oral microbiome than partially and especially totally edentulous ones [13], likely due to the favorable microbial niche provided by the crevicular supra- and sub-gingival areas [14].

Socransky et al. [9] classified the bacteria of the subgingival biofilm community into five main complexes, identified as red, orange, green, yellow, and purple, in 1998. Other microorganisms were also defined as “outliers”, characterized by low relationships with each other and with the bacteria of the five main complexes. Compared to the healthy periodontal sites, periodontitis-associated microbiota is characterized by an increase in both total microbial load and predominant periodontal pathogens, particularly those belonging to the red complex (*Porphyromonas gingivalis*, *Tannerella forsythia*, and *Treponema denticola*), along with *Fusobacterium nucleatum*, *Prevotella intermedia* and *Aggregatibacter actinomycetemcomitans*; a shift from aerobic Gram-negative to strictly anaerobic Gram-negative species is generally observed [15,16].

Similarly, at peri-implant sites, anaerobic bacteria were prevalent, especially when the probing depth (PD) was ≥5–6 mm, thus indicating the need for therapeutic intervention [17], and the predominant species corresponded to periodontal pathogens [18]. However, peri-implantitis-associated microbiota was more heterogeneous [19,20,21] and showed a slightly higher abundance in the red-complex bacteria and, in particular, of the orange complex (in particular, *Prevotella intermedia* and *Prevotella nigrescens*) compared to periodontitis-associated microbiota [12,19,22]. In fact, higher counts of *Prevotella intermedia*, *Prevotella nigrescens*, *Porphyromonas gingivalis*, *Treponema denticola*, *Tannerella forsythia*, and *Aggregatibacter actinomycetemcomitans* were found in peri-implantitis than periodontitis [12,19,22].

Accordingly, the main goals of peri-implantitis treatment include reducing the overall microbial load and reversing dysbiosis, with favorable changes in the peri-implantitis-associated microbiota, and immune-inflammatory modulation of the host [18,19], to achieve healing of the inflamed soft tissue [23], probing depth reduction (≤5 mm), and halting bone loss [4,24], through non-surgical and surgical approaches [25,26,27,28,29] combined or not without adjunctive treatments [4,26,27,30,31,32,33].

The European Federation of Periodontology 2023 clinical practice guidelines for the prevention and treatment of peri-implant diseases [34] recommend that the treatment of peri-implantitis begins with a non-surgical approach. The first reassessment following treatment is recommended 3–4 months after treatment for peri-implantitis, during which time it is recommended to monitor the healing process [34]. Following the reassessment at the third month, based on the outcomes recorded at the reassessment, treatment can continue with a surgical approach or with regular supportive peri-implant care every 3–4 months for at least the first 12 months [34]. Regular supportive peri-implant care is also recommended following surgical treatment and the frequency is every 3–4 months starting from the third month after surgical treatment and for at least the first 12 months [34].

Therefore, the present systematic review of RCTs aimed primarily to characterize the short-term (1-week, 1-month, 3-month follow-up) and long-term (6- and 12-month follow-up) changes in the peri-implantitis-associated microbiota following (any) peri-implantitis treatment in systemically healthy, non-smoking, partially and totally edentulous adult subjects. The secondary aims were to point out the time course of microbial variations in both the biofilm total microbial load and the predominant pathogenic species’ counts at treated peri-implantitis sites and to compare the microbial concentrations and composition with those of healthy peri-implant and periodontal sites and treated peri-implant mucositis, gingivitis, and periodontitis sites.

## 2. Materials and Methods

### 2.1. Study Protocol

The study protocol was developed in accordance with the Preferred Reporting Items for Systematic Reviews and Meta-analyses (PRISMA) statement [22] before performing the electronic and manual literature search, data extraction, and related analysis and was registered on the PROSPERO Registry of Systematic Reviews (number: CRD42024514521).

**Figure 1:**
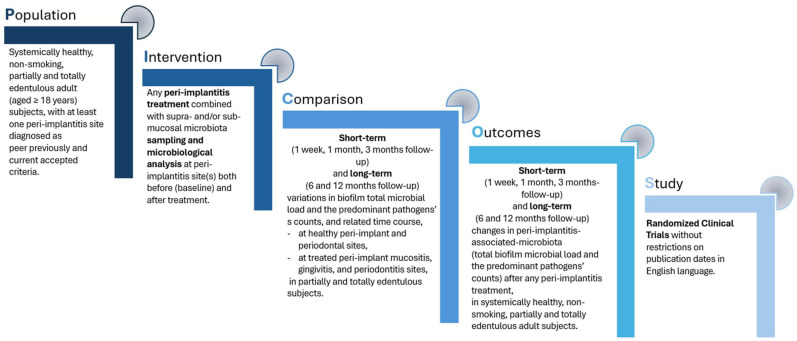
PICO model: Population (P) [3] Intervention (I), Comparison (C), Outcomes (O), Study (s) [36].

The research was carried out to provide current evidence for the following question, developed with the PICOs model [35] (Figure 1): “Which are the short- and long-term changes in the peri-implantitis-associated microbiota in both the total biofilm microbial load and the predominant pathogenic species, following (any) peri-implantitis treatment in systemically healthy, non-smoking, partially and totally edentulous adult subjects?”.

### 2.2. Search Strategy

Two reviewers (C.R.; A.F.) conducted the electronic search independently using pertinent keywords (Table 1), on the MEDLINE/PubMed, Web of Science, and Scopus databases until 5 January 2024 to retrieve English Randomized Clinical Trials (RCTs) without restrictions on publication dates.

The same reviewers (C.R.; A.F.) screened the reference lists of the included studies to retrieve additional potential records.

### 2.3. Study Selection and Eligibility Criteria

Two reviewers (F.D.S.; M.P.D.P.) selected the studies independently of each other. All titles of the records found by the electronic search in the databases and the register were screened, duplicates were eliminated and relevant abstracts were read. A third reviewer (A.R.) was involved in the study selection in case of discrepancies, and all doubts were resolved by discussion. The same two reviewers independently downloaded and reviewed the full texts of the potentially eligible titles/abstracts. The studies’ authors were contacted if the full text was unavailable.

**Figure 2:**
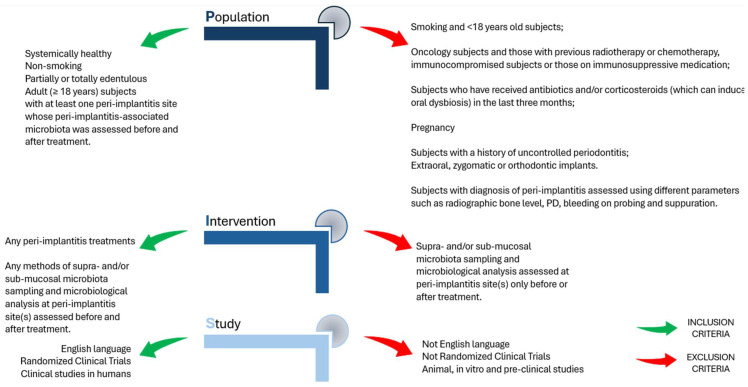
Eligibility (inclusion and exclusion) criteria [3,36].

The same process was performed for records identified by the manual search in the reference list of included studies, applying the same eligibility criteria illustrated in Figure 2.

### 2.4. Data Extraction and Synthesis

Three reviewers (F.D.S.; M.P.D.P.; G.F.) independently performed data extraction and collection using a specific form based on the proposed models for intervention reviews of RCTs [25]. Only data that met the eligibility criteria were extracted and collected. In addition, data from partially edentulous subjects with at least one treated peri-implantitis site were extracted and collected independently from data from totally edentulous subjects (rehabilitated with full-arch dental implant-supported restoration with at least one treated peri-implantitis site), from healthy peri-implant and periodontal sites and from treated peri-implant mucositis, gingivitis, and periodontitis sites.

No attempt was made to contact the Authors of the included studies to obtain or confirm the data.

The following data were extracted and descriptively synthesized from each record using Microsoft Excel software 2019 (Microsoft Corporation, Redmond, WA, USA):

- Studies: authors, year of publication, journal, design of the included studies, quality, funding;
- Population: sample size, mean age, gender ratio; number of peri-implantitis sites treated, number of supported restoration, implant design type, and position;
- Intervention: type and sessions of peri-implantitis treatment, and timing and methods of microbiological sampling and analysis;
- Outcome(s): peri-implantitis-associated microbiota concentration and composition before (at baseline) and at short-term (1 week, 1 month, 3 months) and long-term (6 and 12 months) follow-up after (any) treatment.

### 2.5. Risk Assessment

The RCTs included in the present study were assessed by the toll for evaluating the quality of the systematic reviews of randomized studies: Revised Cochrane Risk-of-Bias 2 tool for randomized trials (RoB 2) [36], accessed freely online (Risk of bias tools—Current version of RoB 2) on 24 February 2024, by three independent reviewers (F.D.S.; M.P.D.P.; G.F.).

The RoB 2 tool takes into account the bias due to the randomization process, the effect of assignment and adhering to intervention, missing data outcome, measurement of outcome, and selection of the reported result [36].

The risk was judged as “low” if the risk of bias was low for all domains; “unclear” if at least one domain was unclear, but no one was high in any domain; “high” if multiple domains were unclear or if a high risk of bias was present in at least one domain [36].

## 3. Results

### 3.1. Study Selection

A total of 1461 records were identified via databases, 341 from PubMed/MEDLINE, 594 from Scopus, and 526 from Web of Science databases; 570 duplicate records were removed. The remaining 891 records were screened by reading the title abstracts, and 713 records were excluded because they were not relevant to the topic of the present systematic review.

All remaining 178 reports sought for retrieval were found without contacting the Authors. The 178 records assessed for eligibility were screened by reading the full text, and 168 articles were excluded for the following reasons: 99 were not RCTs; 30 did not involve subjects with peri-implantitis or peri-implantitis treatment was not performed; 19 because it was not possible to extract data from microbiological analysis or it was not performed; 18 because it was not possible to extract data from non-smoking and/or partially edentulous subjects; 1 because it was not possible to extract data from subjects with peri-implantitis; 1 was not in the English language.

At the end of the study selection identified via databases, 10 RCTs [37,38,39,40,41,42,43,44,45,46] were included in the present systematic review.

The same study selection process was performed by considering the references list of the 10 studies included via the electronic search.

A total of 393 references were identified via manual research; 57 duplicate records were removed. The remaining 336 records were screened by reading the title abstracts, and 324 records were excluded because not relevant to the topic of the present systematic review.

All remaining 12 reports sought for retrieval were found without contacting the Authors. The 12 records assessed for eligibility were screened by reading the full text, and 11 articles were excluded for the following reasons: eight because microbiological analysis was not performed; two were not RCTs; one because it was not possible to extract data from non-smokers.

At the end of the study selection identified via manual search, one RCT [47] was included in the present systematic review.

**Figure 3:**
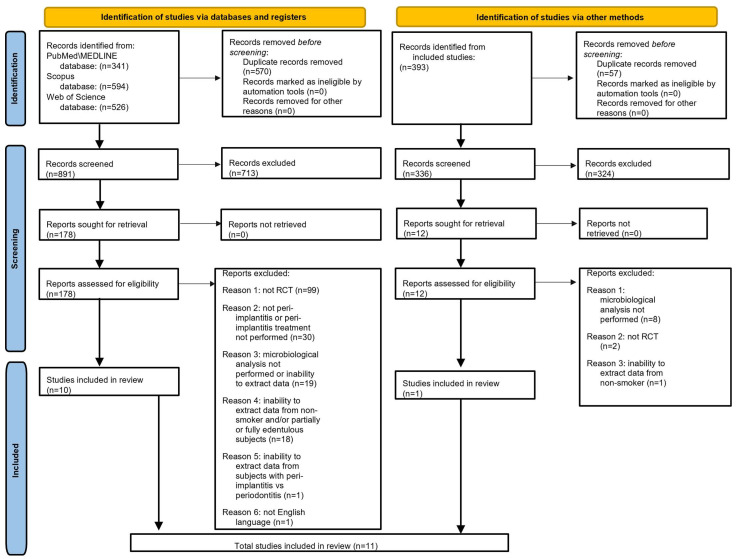
PRISMA 2020 flowchart of the study selection via databases (electronic search) and other methods (manual search).

Finally, 11 RCTs [37,38,39,40,41,42,43,44,45,46,47] were included in the electronic and manual search (Figure 3).

Data from 11 RCTs [37,38,39,40,41,42,43,44,45,46,47] compliant with the eligibility criteria were extracted and synthesized.

No study was found in which the (short- and long-term) concentrations and composition of the peri-implantitis-associated microbiota after (any) peri-implantitis treatment in systemically healthy, non-smoking, partially and totally edentulous adult subjects was examined or in which the data from totally edentulous patients could be extracted independently. Similarly, no data on peri-implantitis-associated microbiota variations were found at healthy peri-implant and periodontal sites and at treated peri-implant mucositis, gingivitis and periodontitis sites.

Table 2 summarizes data from the 11 RCTs included that evaluated the microbiological content of supra- or submucosal samples before and after any peri-implantitis treatment (with or without adjunctive treatment) in systemically healthy non-smoking partially edentulous subjects with at least one dental implant-supported restoration affected by peri-implantitis, diagnosed as peer previously and current accepted criteria [3,17].

Participants’ mean age and gender ratio were specified in six studies [37,39,41,43,44,45] and amounting to 55.62 for the test group and 55.55 for the control group, with a gender ratio M:F of 1.57:1 and 1.05:1, respectively.

No data on totally edentulous adult subjects were retrieved, thus limiting the present microbiological results, which are supposed to be influenced by the coexistence of the periodontal microbial niche, as later explained.

Dental implant characteristics were described in five studies [38,39,40,41,46] concerning the dental implant design, while the type of implant abutment was not reported in any study. The reported implant surface was predominantly sandblasted and acid-etched [38,39,40,41,46].

One study [38] defined the type of prosthesis restoration: cement-retained fixed metal–ceramic (n = 24) in both the test and the control group.

One study [43] specified the total number of prosthesis restorations: single crown (n = 36) and fixed partial prosthesis (n = 64) in both the test and the control group.

One study [39] reported the meantime after implant placement of dental implant treated, which amounted to 7.3 years in the test group and 7.2 years in the control group.

**Figure 4:**
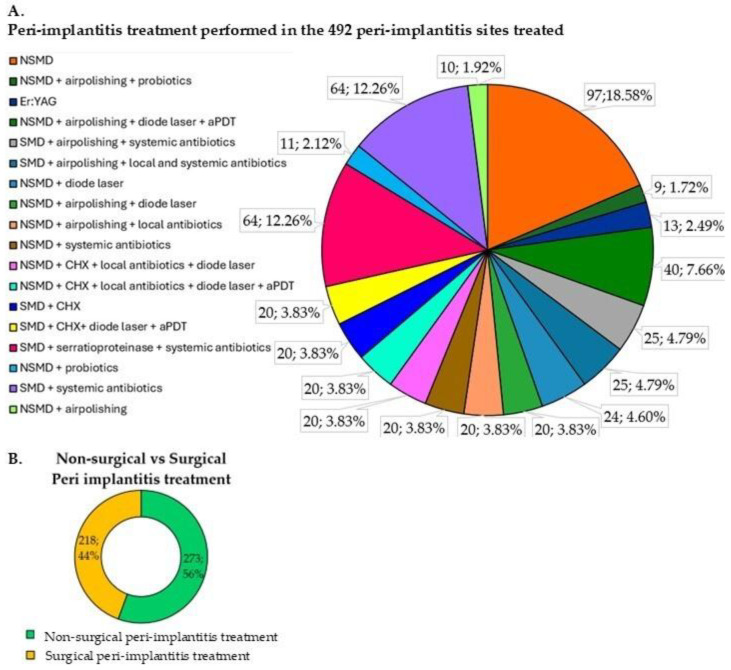
(**A**) Pie chart showing the distribution (numerical and percentage) of peri-implantitis treatment types performed in the 492 peri-implantitis sites treated. (**B**) Pie chart showing the distribution (numerical and percentage) of non-surgical vs. surgical peri-implantitis treatments.

In total, the therapy performed in 273 (56%) peri-implantitis sites was non-surgical treatment, while in 218 (44%) it was surgical (Figure 4).

In two studies [38,45], prosthesis restorations were removed before the peri-implantitis treatment, while one study [40] specified that prosthesis restorations were not removed.

The peri-implantitis treatment was described in six studies as performed in one session [37,38,42,43,44,45], in one study [47] in two sessions, three sessions [42], four sessions [41], five sessions [46], or six sessions [39].

Submucosal sampling was taken in eight studies [38,39,42,43,44,45,46,47], and one of these also performed saliva and tongue sampling [44]. No study reported supra-mucosal sampling.

After peri-implantitis treatment, one study [40] collected the microbiological sampling after treatment; one study [46] after 14 days; two studies after one month [38,43]; one study [44] after six weeks; eight studies after 3 months [39,40,41,42,43,44,46,47]; eight studies after 6 months [37,39,40,41,42,44,45,46]; and three studies after 12 months [37,39,46].

The microorganism identification techniques used were real-time PCR in five studies [39,41,44,45,47], PCR in three studies [37,38,43], bacterial cultures in one study [40], and two studies did not define the technique. DNA was the target of the microorganism identification techniques in five studies [38,39,44,45,46], and 16s rRNA in two studies [44,45].

There were no microbiological data about healthy peri-implant and periodontal sites, and treated peri-implant mucositis, gingivitis, and periodontitis sites, preventing comparison.

### 3.2. Red-Complex Bacteria before and after Peri-Implantitis Treatment

One study [41] reported the percentage of dental implants with peri-implantitis in which the red-complex bacteria load (*Porphyromonas gingivalis*, *Tannerella forsythia*, and *Treponema denticola*) was significant at 3 and 6 months. A decrease was observed both in the test and control group treated with SMD plus air-polishing and local and systemic antibiotics (the test group did not receive local antibiotics).

Another study [46] registered the percentage proportion of red-complex bacteria in submucosal peri-implantitis samples. Both NSMD plus systemic antibiotics and NSMD without adjunctive treatment led to a statistically significant reduction in the percentage proportion of red-complex bacteria at 14 days and 3 months. However, significant regrowth of the percentage proportion of red-complex bacteria was observed from 3 months to 1 year in particular in the NSMD without adjunctive treatment group. At 1 year after peri-implantitis treatment, red-complex bacteria were still in significantly lower percentage proportions in the test group in comparison with baseline, but not in the control group despite multiple NMSD sessions.

#### 3.2.1. *Porphyromonas gingivalis* before and after Peri-Implantitis Treatment

Eight studies [37,38,39,40,41,43,44,47] evaluated the presence and variation of *Porphyromonas gingivalis* before and after peri-implantitis treatment.

One study [38] reported the percentage of dental implants with peri-implantitis in which the *Porphyromonas gingivalis* load was significant at 1 month. No statistically significant decrease was observed in the test group treated with NSMD plus diode laser, while no changes were recorded in the control group treated with NSMD.

One study [39] reported the percentage of dental implants with peri-implantitis in which the *Porphyromonas gingivalis* load was significant at 3, 6, and 12 months. A statistically significant decrease was observed in the test group (treated with NSMD plus air-polishing plus diode laser plus aPDT) and in the control group (treated with NSMD plus air-polishing plus local antibiotics), at any time except at 12 months for the test group.

One study [41] reported that the percentage of dental implants with peri-implantitis, in which *Porphyromonas gingivalis* was significant at 3 and 6 months. Both in the test and control group treated with SMD plus air-polishing and local and systemic antibiotics (the test group did not receive local antibiotics) *Porphyromonas gingivalis* were recorded in significant counts in no peri-implantitis sites at any time.

Five studies [37,40,43,44,47] specified the *Porphyromonas gingivalis* counts after different peri-implantitis treatments and times, as shown in Table S1 in Supplementary File S1.

The microbiological analysis level shows that *Porphyromonas gingivalis* had a greater negative mean absolute deviation from baseline immediately after treatment and thereafter at three months. The greatest increase was recorded after 1 month.

#### 3.2.2. *Tannerella forsythia* before and after Peri-Implantitis Treatment

Six studies [37,38,39,41,43,47] evaluated the presence and variation of *Tannerella forsythia* before and after peri-implantitis treatment.

One study [38] reported the percentage of dental implants with peri-implantitis in which the *Tannerella forsythia* load was significant at 1 month. No statistically significant decrease was observed in the test group treated with NSMD plus diode laser, while no changes were recorded in the control group treated with NSMD.

One study [39] reported the percentage of dental implants with peri-implantitis in which the *Tannerella forsythia* load was significant at 3, 6, and 12 months. A statistically significant decrease was observed in the test group (treated with NSMD plus air-polishing plus diode laser plus aPDT) and in the control group (treated with NSMD plus air-polishing plus local antibiotics), at any time except at 12 months.

One study [41] reported the percentage of dental implants with peri-implantitis in which *Tannerella forsythia* was significant at 3 and 6 months. Both in the test and control group treated with SMD plus air-polishing and local and systemic antibiotics (the test group did not receive local antibiotics) *Tannerella forsythia* was recorded in significant counts in fewer peri-implantitis sites at 3 months, and further decreased at 6 months.

Three studies [37,43,47] specified the *Tannerella forsythia* counts after different peri-implantitis treatments and times, as shown in Table S2 in Supplementary File S1.

The microbiological analysis level shows that *Tannerella forsythia* had a greater negative mean absolute deviation from baseline after six months. The greatest increase was recorded after 1 month.

#### 3.2.3. *Treponema denticola* before and after Peri-Implantitis Treatment

Six studies [37,38,39,41,43,47] evaluated the presence and variation of *Treponema denticola* before and after peri-implantitis treatment.

One study [38] reported the percentage of dental implants with peri-implantitis in which the *Treponema denticola* load was significant at 1 month. No statistically significant decrease was observed in the test group treated with NSMD plus diode laser, while no changes were recorded in the control group treated with NSMD.

One study [39] reported the percentage of dental implants with peri-implantitis in which the *Treponema denticola* load was significant at 3, 6, and 12 months. A statistically significant decrease was observed in the control group (treated with NSMD plus air-polishing plus local antibiotics) at any time, in the test group (treated with NSMD plus air-polishing plus diode laser plus aPDT) at 3 months.

One study [41] reported the percentage of dental implants with peri-implantitis in which *Treponema denticola* was significant at 3 and 6 months. In the test group (treated with SMD, air-polishing and systemic antibiotics) *Porphyromonas gingivalis* was recorded in significant counts in no peri-implantitis sites at any time. In the control group (treated with SMD plus air-polishing, and local and systemic antibiotics) a decrease was found at 3 and 6 months.

Three studies [37,43,47] specified the *Treponema denticola* counts after different peri-implantitis treatments and times, as shown in Table S3 in Supplementary File S1.

The microbiological analysis level shows that *Treponema denticola* had a greater negative mean absolute deviation from baseline after three months. The greatest increase was recorded after 1 month.

**Figure 5:**
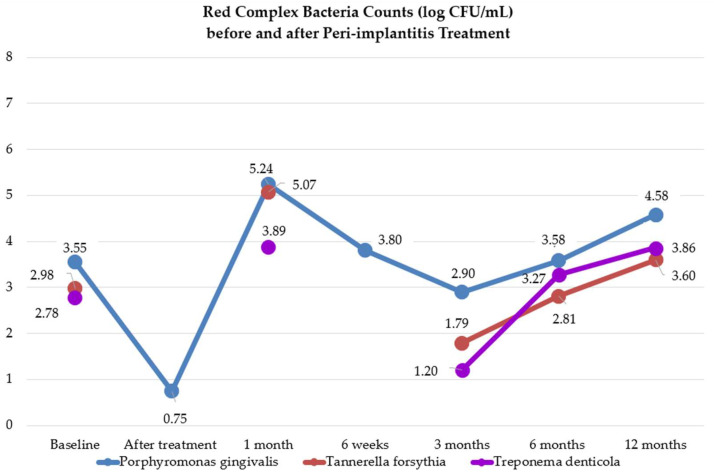
Red-complex bacteria (*Porphyromonas gingivalis*, *Tannerella forsythia*, and *Treponema denticola*) weighted average counts (log CFU/mL) before (baseline) and after (immediately after treatment/1 week, 1 month, 6 weeks, 3 months, 6 months, and 12 months follow-up) treatment.

Figure 5 shows red-complex bacteria count changes before and after peri-implantitis treatments.

### 3.3. Orange-Complex Bacteria before and after Peri-Implantitis Treatment

One study [41] reported the percentage of patients with significant orange-complex bacteria load (*Campylobacter (C.) gracilus*, *C. rectus*, *C. showae Eubacterium nodatum*, *Fusobacterium (F.) nucleatum nucleatum*, *F. nucleatum polymorphum*, *Prevotella interemdia*, *Prevotella nigrescens*, *Peptostreptococcus micros*, and *Streptococcus costellatus*). This percentage remained unchanged both in the test and control group treated with SMD, air-polishing, local and systemic antibiotics (the test group did not receive local antibiotics) both at 3 and 6 months.

Another study [46] registered the percentage proportion of orange-complex bacteria in submucosal peri-implantitis samples. In the test group treated with NSMD plus antibiotics, there was a significant increase in the percentage proportion of orange-complex bacteria at 3, 6 and 12 months. In the control group treated with NSMD without additional therapies, a non-significant reduction was recorded at 3 and 6 months.

#### 3.3.1. *Campylobacter rectus* before and after Peri-Implantitis Treatment

Four studies [38,39,41,43] evaluated the presence and variation of *Campylobacter rectus* before and after peri-implantitis treatment.

One study [38] reported the percentage of dental implants with peri-implantitis in which the *Campylobacter rectus* load was significant at 1 month. No change was observed in the test group treated with NSMD plus diode laser, while statistically significant decreases were recorded in the control group treated with NSMD.

One study [39] reported the percentage of dental implants with peri-implantitis in which the *Campylobacter rectus* load was significant at 3, 6, and 12 months. A statistically significant decrease was observed in the control group (treated with NSMD plus air-polishing plus local antibiotics) at any time.

One study [41] reported the percentage of dental implants with peri-implantitis in which the *Campylobacter rectus* load was significant at 3 and 6 months. In the test group (treated with SMD, air-polishing, and systemic antibiotics) and in the control group (SMD, air-polishing, local and systemic antibiotics), *Campylobacter rectus* was recorded in significant counts in fewer peri-implantitis sites at 3 months, and a further decrease was found at 3 and 6 months.

One study [43] specified the *Campylobacter rectus* counts after different peri-implantitis treatments and times, as shown in Table S4 in Supplementary File S1.

The microbiological analysis level shows that *Campylobacter rectus* had a greater negative mean absolute deviation from baseline after 1 month.

No increase was recorded after peri-implantitis treatment.

#### 3.3.2. *Eubacterium nodatum* before and after Peri-Implantitis Treatment

Three studies [38,39,41] evaluated the presence and variation of *Eubacterium nodatum* before and after peri-implantitis treatment.

One study [38] reported the percentage of dental implants with peri-implantitis in which the *Eubacterium nodatum* load was significant at 1 month. *Eubacterium nodatum* was registered in no peri-implantitis sites in significant load at baseline or after 1 month, except in three subjects in the test group (treated with NSMD plus diode laser) at 1 month.

One study [39] reported the percentage of dental implants with peri-implantitis in which the *Eubacterium nodatum* load was significant at 3, 6, and 12 months. No peri-implantitis sites registered a significant load at any time, both in the test group (treated with NSMD plus air-polishing plus diode laser plus aPDT) and in the control group (treated with NSMD plus air-polishing plus local antibiotics).

One study [41] reported the percentage of dental implants with peri-implantitis in which the *Eubacterium nodatum* was significant at 3 and 6 months. *Eubacterium nodatum* was registered in no peri-implantitis sites in significant load at baseline or after treatment, except in the test group (treated with SMD plus air-polishing and systemic antibiotics) at 3 months.

#### 3.3.3. *Fusobacterium nucleatum* before and after Peri-Implantitis Treatment

Five studies [38,39,41,43,44] evaluated the presence and variation of *Fusobacterium nucleatum* before and after peri-implantitis treatment.

One study [38] reported the percentage of dental implants with peri-implantitis in which the *Fusobacterium nucleatum* load was significant at 1 month. *Fusobacterium nucleatum* was registered in all peri-implantitis sites in significant load in the test group (treated with NSMD plus diode laser) and in the control group (treated with NSMD) at any time.

One study [39] reported the percentage of dental implants with peri-implantitis in which the *Fusobacterium nucleatum* load was significant at 3, 6, and 12 months. A statistically significant decrease was observed in the test group (treated with NSMD plus air-polishing plus diode laser plus aPDT) and in the control group (treated with NSMD plus air-polishing plus local antibiotics), at any time except at 3 months in the test group.

One study [41] reported the percentage of dental implants with peri-implantitis in which the *Fusobacterium nucleatum* was significant at 3 and 6 months. Both in the test and control group treated with SMD plus air-polishing and local and systemic antibiotics (the test group did not receive local antibiotics) *Fusobacterium nucleatum* was registered in all peri-implantitis sites in significant load in the test and control group at any time.

Two studies [43,44] specified the *Fusobacterium nucleatum* counts after different peri-implantitis treatments and times, as shown in Table S5 in Supplementary File S1.

The microbiological analysis level shows that *Fusobacterium nucleatum* had a greater negative mean absolute deviation from baseline after treatment. The greatest increase was recorded after 3 months.

#### 3.3.4. *Peptostreptococcus micros* before and after Peri-Implantitis Treatment

Three studies [38,41,43] evaluated the presence and variation of *Peptostreptococcus micros* before and after peri-implantitis treatment.

One study [38] reported the percentage of dental implants with peri-implantitis in which the *Fusobacterium nucleatum* load was significant at 1 month. No statistically significant decrease was registered in peri-implantitis sites in the test group (treated with NSMD plus diode laser) and in the control group (treated with NSMD).

One study [41] reported the percentage of dental implants with peri-implantitis in which the *Peptostreptococcus micros* load was significant at 3 and 6 months. Both in the test and control group treated with SMD plus air-polishing and local and systemic antibiotics (the test group did not receive local antibiotics) *Peptostreptoccoccus micros* was registered to decrease at 3 months, and no change was recorded between the third and the sixth months.

One study [43] specified the *Peptostreptococcus micros* counts after different peri-implantitis treatments and times, as shown in Table S6 in Supplementary File S1.

The microbiological analysis level shows that *Peptostreptococcus micros* had a greater negative mean absolute deviation from baseline after treatment. No increase was recorded at any time after peri-implantitis treatment.

#### 3.3.5. *Prevotella intermedia* before and after Peri-Implantitis Treatment

Seven studies [38,39,40,41,43,44,47] evaluated the presence and variation of *Prevotella intermedia* before and after peri-implantitis treatment.

One study [38] reported the percentage of dental implants with peri-implantitis in which the *Prevotella intermedia* load was significant at 1 month. An increase was observed in the test group treated with NSMD plus diode laser, and in the control group treated with NSMD.

One study [39] reported the percentage of dental implants with peri-implantitis in which the *Prevotella intermedia* load was significant at 3, 6, and 12 months. A statistically significant decrease was observed in the control group (treated with NSMD plus air-polishing plus local antibiotics) at 3 months.

One study [41] reported the percentage of dental implants with peri-implantitis in which the *Prevotella intermedia* was significant at 3 and 6 months. In the test group (treated with SMD plus air-polishing and systemic antibiotics) and in the control group (treated with SMD plus air-polishing, and local and systemic antibiotics) *Prevotella intermedia* was recorded in significant counts in fewer peri-implantitis sites at 3 months, and a further decrease was found at 3 and 6 months.

Four studies [40,43,44,47] specified the *Prevotella intermedia* counts after different peri-implantitis treatments and times, as shown in Table S7 in Supplementary File S1.

The microbiological analysis level shows that *Prevotella intermedia* had a greater negative mean absolute deviation from baseline immediately after treatment and after three months. The greatest increase was recorded after 1 month.

#### 3.3.6. *Prevotella nigrescens* before and after Peri-Implantitis Treatment

Two studies [38,41] evaluated the presence and variation of *Prevotella nigrescens* before and after peri-implantitis treatment.

One study [38] reported the percentage of dental implants with peri-implantitis in which the *Prevotella nigrescens* load was significant at 1 month. No statistically significant decrease was registered in the *Prevotella nigrescens* load in the test group (treated with NSMD plus diode laser) and in the control group (treated with NSMD).

One study [41] reported the percentage of dental implants with peri-implantitis in which *Prevotella nigrescens* was significant at 3 and 6 months. Both in the test and control group treated with SMD plus air-polishing and local and systemic antibiotics (the test group did not receive local antibiotics), a significant decrease in the *Prevotella nigrescens* load was registered at 3 and 6 months.

#### 3.3.7. *Streptococcus constellatus* before and after Peri-Implantitis Treatment

One study [38] evaluated the presence and variation of *Streptococcus constellatus* before and after peri-implantitis treatment.

One study [38] reported the percentage of dental implants with peri-implantitis in which the *Streptococcus constellatus* load was significant at 1 month. No statistically significant decrease was registered in the *Streptococcus constellatus* load in the test group (treated with NSMD plus diode laser) and in the control group (treated with NSMD).

**Figure 6:**
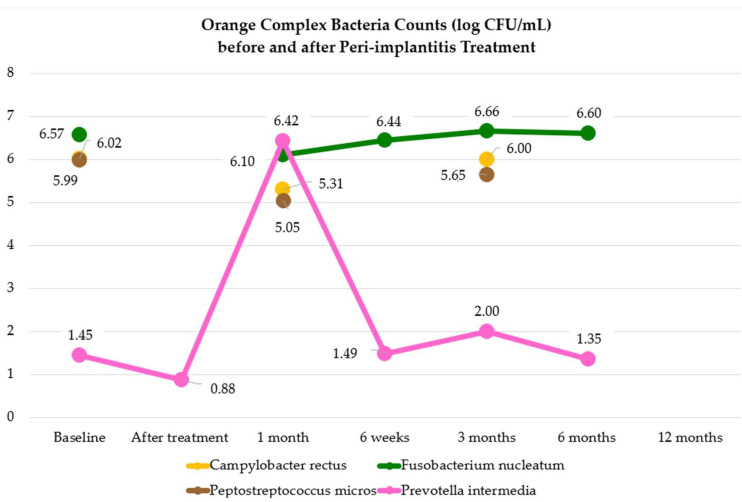
Orange-complex bacteria (*Campylobacter rectus*, *Fusobacterium nucleatum*, *Peptostreptococcus micros*, and *Prevotella intermedia*) weighted average counts (log CFU/mL) before (baseline) and after (immediately after treatment, 1 month, 6 weeks, 3 months, 6 months, and 12 months follow-up) treatment.

Figure 6 shows orange-complex bacteria count changes before and after peri-implantitis treatments.

### 3.4. Green Complex Bacteria before and after Peri-Implantitis Treatment

#### 3.4.1. *Eikenella corrodens* before and after Peri-Implantitis Treatment

Two studies [39,43] evaluated the presence and variation of *Eikenella corrodens* before and after peri-implantitis treatment.

One study [39] reported the percentage of dental implants with peri-implantitis in which the *Eikenella corrodens* load was significant at 3, 6, and 12 months. A statistically significant decrease was observed in the test group (treated with NSMD plus air-polishing plus diode laser plus aPDT) and in the control group (treated with NSMD plus air-polishing plus local antibiotics), at any time.

One study [43] specified the *Eikenella corrodens* counts after different peri-implantitis treatments and times, as shown in Table S8 in Supplementary File S1.

The microbiological analysis level shows that *Eikenella corrodens* had a greater negative mean absolute deviation from baseline after 3 months. No increase was recorded after peri-implantitis treatment.

#### 3.4.2. *Capnocytophaga gingivalis* before and after Peri-Implantitis Treatment

One study [39] evaluated the presence and variation of *Capnocytophaga gingivalis* before and after peri-implantitis treatment.

One study [39] reported the percentage of dental implants with peri-implantitis in which *Capnocytophaga gingivalis* (plus diode laser plus aPDT) and control group (treated with NSMD plus air-polishing plus local antibiotics) was registered in all peri-implantitis sites in significant loads at any time.

### 3.5. Bacteria Outliers from Socransky Complex before and after Peri-Implantitis Treatment

#### 3.5.1. *Aggregatibacter actinomycetemcomitans* before and after Peri-Implantitis Treatment

Five studies [39,40,43,44,47] evaluated the presence and variation of *Aggregatibacter actinomycetemcomitans* before and after peri-implantitis treatment.

One study [39] reported the percentage of dental implants with peri-implantitis in which the *Aggregatibacter actinomycetemcomitans* load was significant at 3, 6, and 12 months. Excluding the baseline, *Aggregatibacter actinomycetemcomitans* was not found at significant levels at any site with peri-implantitis and at any time in both the control (treated with NSMD plus air-polishing plus local antibiotics) and test group (treated with NSMD plus air-polishing plus diode laser plus aPDT).

Four studies [40,43,44,47] specified the *Aggregatibacter actinomycetemcomitans* counts after different peri-implantitis treatments and times, as shown in Table S9 in Supplementary File S1.

The microbiological analysis level shows that *Aggregatibacter actinomycetemcomitans* had a greater negative mean absolute deviation from baseline at one month. The greatest increase was recorded after 6 weeks.

#### 3.5.2. *Parvimonas micra* before and after Peri-Implantitis Treatment

One study [39] evaluated the presence and variation of *Parvimonas micra* before and after peri-implantitis treatment.

One study [39] reported the percentage of dental implants with peri-implantitis in which the *Parvimonas micra* load was significant at 3, 6, and 12 months.

#### 3.5.3. *Pseudomonas aeruginosa* before and after Peri-Implantitis Treatment

One study [45] evaluated the presence and variation of *Pseudomonas aeruginosa* before and after peri-implantitis treatment.

One study [45] reported the percentage of adult subjects with peri-implantitis in which the *Pseudomonas aeruginosa* load was significant at 6 months. In the test group, treated with SMD plus serratiopeptidase and systemic antibiotics, no subjects had a significant load of *Pseudomonas aeruginosa* at 6 months after treatment. In the control group treated with SMD plus systemic antibiotics, a decrease in subjects with a significant load of *Pseudomonas aeruginosa* was observed in both groups at 6 months.

#### 3.5.4. *Staphylococcus aureus* before and after Peri-Implantitis Treatment

One study [45] evaluated the presence and variation of *Staphylococcus aureus* before and after peri-implantitis treatment.

One study [45] reported the percentage of adult subjects with peri-implantitis in which the *Staphylococcus aureus* load was significant at 6 months. In the test group (treated with SMD plus serratiopeptidase and systemic antibiotics) and in the control group (treated with SMD plus systemic antibiotics), a decrease in subjects with a significant load of *Staphylococcus aureus* was observed in both groups at 6 months.

### 3.6. Total Anaerobic Bacteria before and after Peri-Implantitis Treatment

Two studies [40,42] specified the total anaerobic bacteria counts after different peri-implantitis treatments and times, as shown in Table S10 in Supplementary File S1.

The microbiological analysis level shows that the total anaerobic bacteria counts had a greater negative mean absolute deviation from baseline after treatment and thereafter 6 months.

### 3.7. Total Peri-Implant Microbial Load before and after Peri-Implantitis Treatment

One study [43] specified the total peri-implant microbial load at baseline and one and three months after various treatments (Table S11 in Supplementary File S1).

The microbiological analysis level shows that the total bacteria counts had a greater negative mean absolute deviation from baseline after 3 months. The greatest increase was recorded after 1 month.

**Figure 7:**
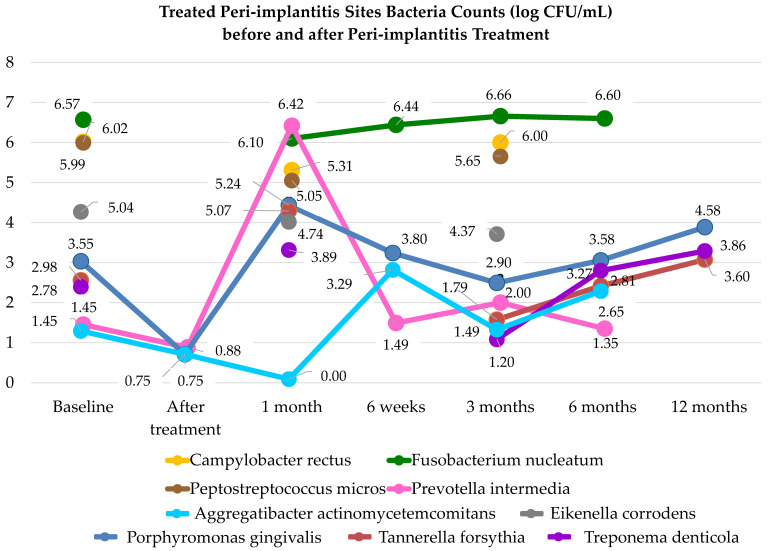
Weighted average counts (log CFU/mL) of the bacteria (Campylobacter rectus, Fusobacterium nucleatum, Peptostreptococcus micros, Prevotella intermedia, Porphyromonas gingivalis, Tannerella forsythia, Treponema denticola, Eikenella corrodens, and Aggregatibacter actinomycetemcomitans) assessed at peri-implantitis sites before (baseline) and after (immediately after treatment/1 week, 1 month, 6 weeks, 3 months, 6 months, and 12 months follow-up) treatment.

Figure 7 summarizes the peri-implantitis-associated microbiota variations after peri-implantitis treatments.

### 3.8. Quality Assessment

The risk of bias and the quality assessment of the RCTs included in the present systematic review were reported in Table S12 and in Figure S1 (Supplementary File S2).

## 4. Discussion

No data on totally edentulous adult subjects were retrieved, thus limiting present microbiological results, which are supposed to be influenced by the coexistence of the periodontal microbial niche. Similarly, there were no data about healthy peri-implant and periodontal sites and treated peri-implant mucositis, gingivitis, and periodontitis sites, preventing results from being compared.

All 11 RCTs [37,38,39,40,41,42,43,44,45,46,47] included in this systematic review were relatively recent (from 2012 to 2022), although the search strategy and eligibility criteria did not include year of publication restrictions. Coherently, only one study [40], published in 2013, used bacterial cultures to identify microorganisms, while most studies described culture-independent techniques. This finding may be attributed to the development of omics technologies (e.g., metatranscriptomics, metaproteomics, metagenomics, and metabolomics) in recent years, which have revolutionized microbiological research and generated particular interest in the study of the human microbiome in health and disease [48,49]. Indeed, these new culture-independent microbiology laboratory techniques, such as next-generation sequencing techniques, have made it possible to define the oral microbiome associated with various health and disease states more accurately and comprehensively [50] and to broaden the microorganism spectrum previously identifiable only by culture-dependent techniques [51]. Nevertheless, the timing of microbiological analysis was extremely heterogeneous in the different studies, jeopardizing data.

The study population, consisting of 432 systemically healthy, non-smoking, partially edentulous subjects with 492 treated peri-implantitis sites, may seem modest, especially when compared to the estimates of dental implants placed annually and considering that peri-implantitis is the most common late complication [52], affecting approximately 15–57% of subjects and 8–28% of implants [15]. However, the combination of supra- and/or submucosal microbiota sampling and microbiologic analysis of the peri-implantitis site(s) both before (baseline) and after (different time points) treatment should be considered and the retrieved findings can still provide a comprehensive overview.

Although not confirmed by clinical evidence, as stated by the 6th European Association for Osseointegration (EAO) Consensus Conference in 2021 [53], peri-implantitis-associated microbiota have been linked to the material, implant design [54], and surface characteristics of dental implants [16,17,18,19], at least from preclinical experimental studies on the progression of untreated peri-implantitis.

Specifically, peri-implant biofilm was proposed to be influenced by the dental implant abutment material [55], while none of the included studies validated this hypothesis. As also revealed from the present results, Titanium (alloys) is the most commonly used material for fabricating dental implant abutments [56], despite the growing demand for more esthetic restorations, which has led to an increasing use of ceramics or polymers [57]. Nevertheless, Del Rey et al. [57], investigating variations in peri-implant biofilm formation on different abutment materials under oral conditions, found no significant microbiological differences in their systematic review [57].

Moreover, only four studies [38,39,40,41,46] outlined the design characteristics of dental implants, all of which featured a rough surface predominantly achieved through sandblasting and acid etching. It is widely acknowledged that a rough surface on dental implants facilitates bacterial adhesion and colonization, unlike smoother surfaces [58,59,60]. Specifically, surface roughness was shown to significantly influence the initial stages of peri-implant biofilm formation, namely adhesion and colonization [59,61], while contrasting results were obtained for the later stages of peri-implant biofilm maturation [58,59,60]. In fact, some studies showed an increased microbial load and higher counts of pathogenic bacteria in mature per-implant biofilm on rougher dental implant surfaces [58,60], while other studies found no differences in biofilm composition in the later compared to the early stage of the peri-implant biofilm formation [59].

Moreover, a comparable distribution was observed between NSMD (56%) and SMD (44%) peri-implantitis treatments, as shown in Figure 4B, when considering the included RCTs’ test and control groups together. NSMD alone emerged as the most prevalent treatment modality and was frequently employed across various studies, primarily within the control group. Nevertheless, despite the considerable heterogeneity in adjunctive treatments, the notable proportion of SMD proved a homogeneous view of both approaches regarding microbiological implications.

Furthermore, none of the studies encompassed resective or regenerative surgical procedures among SMD approaches to treating peri-implantitis. These surgical procedures are typically indicated for horizontal or single-walled non-containable bone defects and vertical or two/three-walled containable defects, respectively [62,63,64,65,66,67]. Their absence in the RCTs examined is notable as these procedures have the potential to abruptly and significantly alter the anatomy of the peri-implantitis site [68], which in turn can change the microbiological load and composition [62] faster than the other SMD and NSMD procedures.

### 4.1. Microbiological Analysis of the Peri-Implantitis-Associated Microbiota

#### 4.1.1. Microbiological Analysis at Baseline before Peri-Implantitis Treatments

All 11 RCTs [37,38,39,40,41,42,43,44,45,46,47] included in the present systematic review had reported the microbiological analysis finding at baseline before peri-implantitis treatments.

*Fusobacterium nucleatum*, *Prevotella intermedia*, as well as *Campylobacter rectus* and *Peptostreptococcus micros*, belonging to the orange complex, were the most prevalent bacteria found in the submucosal samples from peri-implantitis sites at baseline [69]. This finding aligns with previous evidence that some orange-complex bacteria, such as *Fusobacterium nucleatum* and *Prevotella intermedia*, prevail in peri-implantitis sites compared to healthy peri-implant ones [70].

Anaerobic bacteria of the red complex, which includes *Porphyromonas gingivalis*, *Treponema denticola*, and *Tannerella forsythia*, were associated with peri-implantitis disease [71,72], hypothesized because changes in oxygen tension and nutrient concentration are associated with increasing pocket depth and could be responsible for the microbiologic shift [71], and were found second most frequently in the submucosal peri-implantitis samples before treatment. Conversely, red-complex bacteria were recognized as the leading species in periodontitis progression [71]. This observation may rely on the fact that peri-implantitis and periodontitis present distinct microbial ecosystems, each uniquely shaping the quantitative and qualitative composition of their resident microbiota, with limited influence from neighboring niches. Notably, peri-implant sites tend to harbor a less diverse microbiota than periodontal sites, irrespective of their health or disease status. Nevertheless, certain bacterial taxa, such as staphylococci, appear particularly characteristic of the peri-implant niche, and evidence indicates that the microbiota associated with peri-implant sites tends to increase in complexity as the infection progresses from peri-implant mucositis to peri-implantitis. Specifically, peri-implant mucositis is believed to play a significant role in infection advancement, often displaying elevated levels of periodontal pathogens, which may contribute to establishing a microbiota associated with a heightened risk of harm [73].

As for the outlier bacteria from the Socransky complexes, an earlier study found a tendency for the association of *Aggregatibacter actinomycetemcomitans* and peri-implantitis, even if it did not reach a statistically significant level [74]. In contrast to peri-implantitis, the role of *Aggregatibacter actinomycetemcomitans* in the previously classified juvenile and localized aggressive periodontitis is well-established [75]. Indeed, the bacterium is most closely associated with the progression of periodontitis, particularly in aggressive forms [75]. However, in the present systematic review, equal levels of *Aggregatibacter actinomycetemcomitans* and *Prevotella intermedia* were found at baseline.

None of the studies in the present systematic review recorded the presence or change of *Staphylococcus epidermidis*. However, for the first time, Carvalho et al. [74] reported its strongest association with peri-implantitis. The authors suggested that *Staphylococcus epidermidis*, which can only colonize the peri-implant tissues and not the dental implant surface, was found in the biofilm that was free of suppuration when probing peri-implantitis sites with planktonic infections [74]. This could explain the lack of evidence in our samples, which were all taken from the submucosal biofilm.

#### 4.1.2. Microbiological Analysis after Peri-Implantitis Treatment

The results of the present systematic review highlight a marked decrease in *Porphyromonas gingivalis*, *Prevotella intermedia*, and *Aggregatibacter actinomycetemcomitans* when samples were taken immediately after mechanical treatment of peri-implantitis. This finding, evaluated in one study [40], could be related to the fact that both NSMD and SMD approaches are able to effectively remove the peri-implant biofilm, as expected, and thus reduce the microbial load at the peri-implant site, independently of adjunctive therapy.

Indeed, in all studies and both test and control groups, NSMD or SMD were performed alone or in combination with adjunctive treatments, except for 13 peri-implantitis sites that were treated with Er:YAG alone [42]. In the latter cases, the peri-implant biofilm was effectively removed by the use of Er:YAG alone without a significant increase in the dental implant surface temperature, although the same authors recorded better biofilm removal when Er:YAG was combined with NSMD, also compared to NSMD alone [76].

#### 4.1.3. Microbiological Analysis at 1 Month after Peri-Implantitis Treatment

Two RCTs [38,43] included in the present systematic review reported the microbiological analysis finding one month after peri-implantitis treatment.

As conceivable, mechanical debridement, either alone or in combination with additional treatments, does not eradicate the microorganisms at the peri-implant sites, which are, in any case, expected to be recolonized [77]. In detail, in partially edentulous adult patients, crevicular and subgingival areas of natural teeth have been proposed to play a role as a microbial reservoir for recolonizing the submucosal area around dental implants [77]. In addition, peri-implant pockets were found to be colonized with red and orange-complex bacteria associated with periodontitis after only one week. This was demonstrated by Quirynen et al., who compared submucosal biofilm samples collected around dental implants over time with subgingival biofilm samples from the same subjects [78].

Accordingly, one month after (any) peri-implantitis treatment, the submucosal area was presently found to be recolonized by the red and orange-complex bacteria [78]. Specifically, *Porphyromonas gingivalis* and *Tannarella forsythia* of the red complex, *Prevotella intermedia*, *Fusobacterium nucleatum,* and *Campylobacter rectus* of the orange complex, along with *Peptostreptococcus micros*, were predominant compared to the other species in peri-implantitis sites of partially edentulous patients, one month after treatment. Since Quirynen et al. [78], similarly detected *Prevotella intermedia*, *Fusobacterium nucleatum* and *Peptostreptococcus micros* in the majority of submucosal biofilms collected from healthy peri-implant sites of partially edentulous subjects early after implant placement, it may be hypothesized that these bacteria should be considered early colonizers of both healthy and peri-implantitis dental implant pockets.

In detail, except for *Prevotella intermedia*, bacteria of the orange complex, which were more representative of the submucosal biofilm in the peri-implantitis site before treatment, appeared to decrease, although slightly, at one month of peri-implantitis treatment compared to the baseline.

Conversely, and remarkably, *Prevotella intermedia* from the orange complex, as well as *Porphyromonas gingivalis*, *Tannerella forsythia*, and *Treponema denticola* from the red complex, were even elevated one month after peri-implantitis treatment when compared to baseline.

This observation may be due, on the one hand, to the persistence or only discrete improvement of pathological conditions favoring dysbiosis in the peri-implant tissues treated one month earlier and, on the other hand, to the general decrease in the microbial load after treatment, coupled with the specific metabolic characteristics and virulence factors, including the adhesion and invasion capabilities of these bacteria, which unexpectedly increased one month after treatment.

Indeed, it must be considered that none of the studies included in this systematic review performed resective or regenerative bone surgical treatments, which are associated with a decrease in peri-implant pocket depth in a shorter time with rapid modifications in microbiological load and composition [62], as already mentioned. As a counterpart, following other SMD and all NSMD approaches, the progressive reduction in peri-implant pocket dept is expected to take more than three weeks after the restoration of biofilm control [79]. Therefore, oxygen tensions are still likely to be low in peri-implantitis sites after one month of peri-implant treatment, continuing to favor the dysbiotic microbiologic shift, anaerobic species, and red-complex bacteria, along with *Prevotella intermedia* [73].

In addition, it may also be hypothesized that peri-implantitis treatment, controlling the biofilm with a general reduction of the total microbial load [40,42,43], reduces the bacterial species that compete with the red complex ones for the niche of the peri-implant site [40,43,80], further favoring those with higher virulence, capable of recolonizing the treated peri-implantitis sites more quickly at the expense of the less virulent ones [39,43]. It can, therefore, be assumed that the peri-implant biofilm may act as a protective, metabolically active, and dynamically organized microbial community that competes with, and thus balances, the predominant pathogens [74,81], exerting a kind of “competitive balancing effect”. Accordingly, based on the microbiological findings recorded one month after the treatment [39,43], the qualitative and quantitative reduction of the peri-implant biofilm subsequent to peri-implantitis treatment [40,42,43] and the consequent loss of its “competitive balancing effect” allowed the red-complex bacteria and *Prevotella intermedia*, endowed with greater virulence [82], ability to invade host cells and obligate anaerobic metabolism [82], to faster recolonize the peri-implant niche at the expense of the other bacteria of the orange complex and biofilm community species [39,43].

Furthermore, both surgical and non-surgical treatment approaches for peri-implant mechanical debridement in combination with various chemical and physical adjunctive treatments may still fail to have a significant impact on tissue-invasive bacterial species that may persist locally [83].

Indeed, *Prevotella intermedia*, an obligate anaerobic Gram-negative bacterium, is classified according to its fimbrial diameter. *Prevotella intermedia* 17 has type C fimbriae, not found in other strains, such as *Prevotella intermedia* 27, which has type D, and *Prevotella intermedia* 25611 type A, respectively [82]. The type C fimbriae and the cytoskeletal rearrangement of *Prevotella intermedia* 17 have been associated with the significantly greater ability of *Prevotella intermedia* 17 to be internalized in the oral epithelial cells compared to the other *Prevotella intermedia* strains [82]. As invasion is an important step in the infection process, the differentiation of *Prevotella intermedia* strains found in peri-implantitis sites may be important to understand the results of the present study, which showed that the overall highest deviation from baseline was found after one month for *Prevotella intermedia*.

*Porphyromonas gingivalis*, an anaerobic Gram-negative bacterium, is also able to invade gingival epithelial cells to evade the immune system and replicate [84]. This property is associated with its large fimbriae that, after binding to the β1 integrin on the surface of the host cells, cause a rearrangement of the actin cytoskeleton bridges to allow internalization [84]. Once *Porphyromonas gingivalis* has invaded host cells, it secretes an ATP-hydrolyzing enzyme to prevent cell apoptosis or necrosis and allow its intracellular survival [84]. In addition, it can spread from cell to cell without inducing cell death and spread by evading the immune system [84].

*Tannerella forsythia*, an anaerobic Gram-negative bacterium, is able to adhere to and invade host cells due to its fimbriae and surface glycoproteins, which enable adhesion to lectin-like receptors of host cells and subsequent invasion [85]. The lectin-like receptor is also present on *Fusobacterium nucleatum*’s surface, with *Tannerella forsythia* having an affinity to form coaggregation in biofilms [85].

*Treponema denticola*, an obligate anaerobic Gram-negative bacterium, does not possess specific adhesion structures such as fimbriae [86]. Its ability to adhere to host cells is limited by unspecific adhesion factors present on the microorganism’s surface [35]. However, *Treponema denticola* registered the lowest positive mean deviation at one month compared to the baseline [86].

#### 4.1.4. Microbiological Analysis at 3 Months after Peri-Implantitis Treatment

Eight RCTs [39,40,41,42,43,44,46,47] included in the present systematic review reported the microbiological analysis finding three months after peri-implantitis treatment.

Three months after peri-implantitis treatment, bacteria of the orange complex, except *Prevotella intermedia*, were found in larger quantities than after one month. Therefore, with due exception, microbiological data from peri-implantitis sites treated three months before were retraced from the submucosal sampling approximately three months after implant placement which revealed an enlargement of red and orange bacteria compared to after one month [78].

Conversely, the bacteria of the red complex, particularly *Porphyromonas gingivalis*, were found in lower quantities compared with after one month, probably due to clinical improvements with probing depth reduction at the peri-implant sites treated three months before.

In addition, a comparison of the bacterial counts between the initial value and three months after treatment showed that the microbiological values measured after three months leveled off, except for the bacteria of red complex, *Prevotella intermedia* and *Aggregatibacter actinomycetmcomitans*, which instead recorded a decrease of more than one unit. In fact, for the other bacteria species, the values at the beginning of the study did not differ by more than one unit from the values after three months.

#### 4.1.5. Microbiological Analysis at 6 and 12 Months after Peri-Implantitis Treatment

Eight [37,39,40,41,42,44,45,46] and three RCTs [37,39,46] included in the present systematic review reported the microbiological analysis findings at six and twelve months, respectively.

The microbiological analysis data after twelve months were only found for the microorganisms of the red complex, while after six months data were also recorded for *Fusobacterium nucleatum*, *Prevotella intermedia* and *Aggregatibacter actinomycetemcomitans*, but not for all other bacteria of the orange complex.

Analyzing the development of the microorganisms recorded after six and twelve months, a clear leveled off in bacterial species can be seen after six months, and then increased after twelve months compared to the baseline. To deepen these fluctuations, it is important to remember that in most of the included studies, further peri-implantitis treatments were performed after the sixth month. Therefore, a leveling off in the analyzed bacterial species can be observed after six months, despite the retreatment sessions, which consisted of the NSMD regardless of treatment type performed at baseline.

After twelve months, however, the red complex bacteria increased again due to the new reformation of peri-implant biofilm. Interestingly, after twelve months (i.e., six months after retreatment), the red-complex bacterial species, which were the only ones analyzed at that time, returned to levels comparable, but slightly higher, to baseline. This result is consistent with the Hakkers et al. [87] study, in which 25 subjects with peri-implantitis refractory to non-surgical treatments were treated with resective surgery. Although the authors performed maintenance therapy at three, six, and nine months, they found one month after the resective surgery that the reduction in peri-implant microbial load after maintenance therapy was transient and returned to baseline levels after twelve months [87].

#### 4.1.6. *Fusobacterium nucleatum* the “Outlier of the Biofilm Competitive Balancing Effect”

Five studies [38,39,41,43,44] included in the present systematic review evaluated the presence and variation of *Fusobacterium nucleatum* before and after peri-implantitis treatment.

A separate mention must be addressed to *Fusobacterium nucleatum*, whose prevalence remained almost unchanged at any time after treatment, probably due to the ability of the bacterium to invade peri-implant tissues [88], rapidly recolonize treated sites, and not respond to most peri-implantitis treatments.

*Fusobacterium nucleatum*, an anaerobic Gram-negative bacterium, is one of the most prevalent oral and periodontal species, both in health and disease [89], and plays an important role in biofilm formation as a bridging microorganism linking the early colonizers to the anaerobic secondary colonizers [88]. In addition to promoting coaggregation between different bacterial species, *Fusobacterium nucleatum* is also capable of directly invading epithelial and endothelial host cells [89]. Adherence and invasion are important mechanisms for the colonization and dissemination of *Fusobacterium nucleatum* and evasion of the immune system [89]. Indeed, recent evidence has emphasized its ability to evade the immune system response and resist therapies aimed at treating oral pathologies, inflammatory disorders, and neoplasms far from the oral cavity [90,91].

*Fusobacterium nucleatum* also proved to be the most resistant bacterium to tetracycline, metronidazole, erythromycin, and clindamycin administered for the treating peri-implantitis, probably due to its ability to mediate chemoresistance by modulating autophagy, as demonstrated in squamous cell carcinoma of the esophagus [90].

The microbiological analyses reported in the present systematic review showed a greater reduction in *Fusobacterium nucleatum* concentrations at peri-implantitis sites registered after NSMD plus probiotics compared to other treatments. Accordingly, in studies where probiotics were used as an adjunct to periodontal treatment, *Lactobacillus reuteri* was found to be able to reduce periodontal pathogens, such as *Aggregibacter actinomycetemcomitans*, *Prevotella intermedia*, *Fusobacterium nucleatum*, and *Porphyromonas gingivalis* [92]. Similarly, Haukioja et al. [93] found a reduction in periodontal pathogens in the gingival biofilm due to the coaggregation of *Fusobacterium nucleatum* and *Bifidobacterium*.

In summary, peri-implantitis treatment, whether surgical or non-surgical, can effectively reduce the total microbial load, but may also lead to shifts in the bacterial composition over time, due to the peri-implant biofilm nature of a dynamically and metabolically active organized microbial community. The rapid recolonization of peri-implant sites by the more virulent and invasive bacteria is probably associated with the inability of peri-implantitis treatment to eradicate the bacteria characterized by tissue-invasive properties, which were the earlier to recolonize the peri-implant sites.

In addition, it must be considered that none of the studies included in this systematic review performed resective or regenerative bone surgical treatments, which are associated with a decrease in peri-implant pocket depth in a shorter time with rapid modifications in microbiological load and composition. Instead, after non-surgical or surgical treatment without resective or regenerative procedures, the low oxygen tensions re-established in deep peri-implantitis pockets before their physiological healing favor the recolonization of the peri-implant sites by anaerobic bacterial species, which by their tissue-invasive nature, are also the most resistant to peri-implantitis treatment.

Immediately after peri-implantitis treatment, although, as expected, complete eradication has not been recorded, the peri-implant biofilm goes through a reduction of the total microbial load, and of the bacterial species that compete with the red complex.

Recordings after one month show recolonization of the peri-implant site, albeit with a different biofilm composition than at baseline. At one month of peri-implantitis treatment, except for *Prevotella intermedia*, the bacteria of the orange complex which were more representative of the submucosal biofilm in the peri-implantitis site before treatment, appeared to decrease, although slightly, compared to the baseline. Conversely, *Prevotella intermedia* and the red complex bacteria were elevated compared to the baseline.

Three months after peri-implantitis treatment, except *Prevotella intermedia*, bacteria of the orange complex, were found in larger quantities than after one month. Conversely, *Prevotella intermedia* and the bacteria of the red complex, particularly *Porphyromonas gingivalis*, were found in lower quantities, probably due to clinical improvements with probing depth reduction at the peri-implant sites treated three months before. In addition, a comparison of the bacterial counts between the initial value and three months after treatment showed that the microbiological values measured after three months leveled off, except for the bacteria of red complex, *Prevotella intermedia* and *Aggregatibacter actinomycetmcomitans*.

Analyzing the development of the microorganisms recorded after six and twelve months, a clear leveling off in bacterial species can be seen after six months. Interestingly, after twelve months, the red-complex bacterial species, which were the only ones analyzed at that time, returned to levels comparable, but slightly higher, to baseline.

A separate mention must be addressed to *Fusobacterium nucleatum*, whose prevalence remained almost unchanged at any time after treatment, probably due to the ability of the bacterium to invade peri-implant tissues, rapidly recolonize treated sites, and not respond to most peri-implantitis treatments.

### 4.2. Strengths, Limitations, and Future Prospectives

To the best of our knowledge, the present systematic review represents the pioneering effort to characterize short- and long-term changes in peri-implantitis-associated microbiota subsequent to peri-implantitis treatment and offers valuable insights into the temporal dynamics of total biofilm microbial load reduction and fluctuations in predominant pathogenic species concentrations in treated peri-implant sites, thereby enriching comprehension of this multifaceted issue.

All RCTs presently included [37,38,39,40,41,42,43,44,45,46,47] were published relatively recently (from 2012 to 2022), notwithstanding the absence of constraints on publication year. Additionally, most studies employed culture-independent techniques, such as next-generation sequencing, thus broadening the spectrum of microorganisms previously detectable solely through culture-dependent methods [50].

Moreover, the exclusion of medically compromised subjects has mitigated, if not entirely eliminated, potential confounders associated with disorders themselves or medications on peri-implant tissue status, healing, and biofilm accumulation and composition [7,94]. Similarly, the exclusion of traditional tobacco, heat-not-burn, and vapor smokers has removed the influence of nicotine, tobacco, heat, and other substances associated with these habits on peri-implant tissue health, blood perfusion, and biofilm composition [95,96].

Furthermore, the comparable distribution of non-surgical (56%) and surgical (44%) peri-implantitis treatments, and the absence of studies encompassing resective or regenerative surgical approaches to peri-implantitis treatment—approaches capable of abruptly and significantly altering peri-implantitis site anatomy [97], thereby potentially affecting microbiological load and composition more rapidly than other techniques—have provided a uniform perspective for both treatment modalities regarding microbiological implications.

However, microbiological samples from peri-implantitis sites were collected at varying follow-up intervals across studies, precluding parallel treatment comparisons. Moreover, the lack of standardized follow-up intervals registered in the included studies created heterogeneity in the data investigated at each time point, allowing for qualitatively synthesized data, but given their heterogeneity precluding parallel treatment comparisons. Additionally, the methodological heterogeneity of microbiological analyses, including the types of microorganisms investigated and the units of measurement utilized, hindered quantitative analysis of results.

In addition, none of the eleven RCTs reviewed [37,38,39,40,41,42,43,44,45,46,47] specifically collected relevant data from totally edentulous subjects or differentiated them from partially edentulous subjects, thereby impeding distinctive characterization and comparison. Nonetheless, considering that subgingival biofilm in partially edentulous subjects with dental implants influences microbial colonization of dental implants [98] and that the full mouth extraction procedure is associated with a reduction in predominant periodontal pathogens over time [99], future studies should characterize peri-implantitis-associated microbiota before and after treatment in totally edentulous subjects, and compare supra- and submucosal microbial profiles with those from partially edentulous subjects.

Additionally, delving into the functional and virulence distinctions among species strains may directly impact the pathogenicity of the entire microbial community—an area yet to be extensively explored in peri-implant infections. Researching the ecological triggers of functional pathogenicity shows promise in refining strategies for risk assessment, prevention, diagnosis, and supportive therapy. Although metatranscriptomic pathways specific to peri-implantitis were identified, the feasibility of chair-side detection remains challenging. Nevertheless, technological advancements offer the potential for targeted interventions against taxa identified as differentially abundant in peri-implantitis or even in the early stages of peri-implant mucositis. Preliminary data suggest that specific oxidoreductase or complement pathway inhibitors could hold therapeutic potential [73].

## 5. Conclusions

Microorganisms were not eradicated by mechanical debridement, whether used alone or with supplementary treatments, and recolonization of treated peri-implantitis sites is expected regardless. However, the peri-implant total biofilm, as well as the red and orange complex bacteria, was controlled for up to approximately three months immediately following both surgical and non-surgical approaches.

*Prevotella intermedia*, *Fusobacterium nucleatum*, and *Peptostreptococcus micros* were detected in the majority of submucosal biofilms collected immediately after treatment, as well as from healthy peri-implant sites shortly after implant placement, suggesting that these bacteria may act as early colonizers in both peri-implant healthy and peri-implantitis sites.

Despite treatment, *Porphyromonas gingivalis*, *Tannerella forsythia* and *Treponema denticola* from the red complex, and *Prevotella Intermedia* from the orange complex, increased one month after treatment compared to baseline.

While red-complex bacteria decreased at the 3-month follow-up, their levels gradually returned to baseline by six months, and by twelve months, increased again, likely due to the reformation of the peri-implant biofilm.

**Clinical Significance:**
  - Mechanical debridement, whether used alone or in conjunction with supplementary treatments, failed to eradicate microorganisms from peri-implant sites, which are likely to be recolonized regardless.
  - Immediately following both surgical and non-surgical approaches, the total peri-implant biofilm, as well as the red and orange complex bacterial load, was controlled for up to three months. However, the microbiological values of red-complex bacteria measured at six months gradually returned to baseline.
  - Rigorous supportive care and maintenance protocols, including professional mechanical debridement, may be recommended even shortly after peri-implantitis treatment to control red-complex bacterial levels for up to three months.
  - Innovative therapeutic strategies should be considered to effectively manage and target persistent peri-implantitis pathogens.

## Figures and Tables

**Table 1:** Search strategy and filters used for each database.

Search strategy #1 AND #2 AND #3	#1: “peri-implantitis” OR “peri-implant failure” OR “peri-implant disease” OR “implant failure”		
	#2: microbiota OR microbiome OR bacteria OR virus OR viruses OR fungi OR fungus OR microorganisms OR “oral dysbiosis”		
	#3: treatment OR approach OR approaches OR therapy OR “non-surgical treatment “ OR “surgical treatment” OR debridement		
	Databases		
	MEDLINE/PubMed	Web of Science	Scopus
Filters	“Article language”: English.	“Languages”: English.	“Languages”: English.
	“Article type”: not review; not systematic review.	“Document type”: article.	“Document type”: article.

**Table 2:** Studies characteristics and data extracted from the included RCTs. Studies characteristics: first Author, years of publication, journal, design, reference, quality assessment, and funding. Characteristics of the test/control group population: participants’ sample size (n.), mean and range age, gender ratio, implant with and without peri-implantitis (n.), implant with peri-implantitis (n.), implant design and type, type of abutment and prosthesis, supported restoration, position (tissue/bone level), mean time after implant placement. Intervention in the test/control group: peri-implant treatment, removal of the prosthesis (yes/no), session (n.), type of samples(s) (supra- or submucosal), method(s) of sampling collection, timing of collection after treatment, microorganism identification technique, target. Outcome(s): microorganism detected before and after peri-implantitis treatment.

Studies	Test Group	Intervention	Outcome(s) Microorganisms Detected		Control Group	Intervention	Outcome(s) Microorganisms Detected	
			Before Intervention	After Intervention			Before Intervention	After Intervention
Almohareb T., 2020 *Photodiagnosis Photodyn Ther* [37] RCT High risk Deanship of Scientific Research, King Saud University	Test group: n.20 Mean age: 51.7 ± 7.5 y.o. Gender ratio: 18M/2F Implant: n.43—with peri-implantitis: n.20 Implant design and type: MD Type of abutment: MD Type of prosthesis: MD Supported restoration: MD Position: MD Mean time after implant placement: MD	Treatment: NSMD + local antibiotics (AMX 500 mg/3 d + MTZ 400 mg/7 d) + 0.12% CHX + diode laser + aPDT Removal of the prosthesis: MD Session: n.1 Type of sample(s): MD Method(s) of sampling collection: paper points Timing of collection after treatment: 6 and 12 months Microorganism identification technique: PCR Target: MD	*Porphyromonas gingivalis* (log CFU/mL)		Control group: n.20 Mean age: 50.9 ± 6.3 y.o. Gender ratio: 16M/4F Implant: n.36—with peri-implantitis: n.20 Implant design and type: MD Type of abutment: MD Type of prosthesis: MD Supported restoration: MD Position: MD Mean time after implant placement: MD	Treatment: NSMD + local antibiotics (AMX 500 mg/3 d + MTZ 400 mg/7 d) +0.12% CHX + diode laser Removal of the prosthesis: MD Session: n.1 Type of sample(s): MD Method(s) of sampling collection: paper points Timing of collection after treatment: 6 and 12 months Microorganism identification technique: PCR Target: MD	*Porphyromons gingivalis* (log CFU/mL)	
			5.73 ± 1.12	At 6 months 3.24 ± 1.52 *^,‡^			5.29 ± 1.64	At 6 months 3.96 ± 1.11 *
				At 12 months 4.67 ± 1.44				At 12 months 4.48 ± 1.35
			*Tannerella forsythia* (log CFU/mL)				*Tannerella forsythia* (log CFU/mL)	
			4.22 ± 1.73	At 6 months 2.64 ± 1.23 *			4.46 ± 1.21	At 6 months 2.98 ± 1.18 *
				At 12 months 3.33 ± 1.74				At 12 months 3.86 ± 1.89
			*Treponema denticola* (log CFU/mL)				*Treponema denticola* (log CFU/mL)	
			4.19 ± 1.92	At 6 months 3.12 ± 1.09			4.54 ± 1.08	At 6 months 3.41 ± 0.89 *
				At 12 months 3.75 ± 1.79				At 12 months 3.96 ± 1.88
Arısan V., 2015 *Photomed Laser Surg* [38] RCT High risk Istanbul University Research Fund	Test group: n.5 Mean age: N/D Gender ratio: N/D Implant: n.48—with peri-implantitis: n.24 Implant design and type: n.24 tapered root form design with rough surface (sandblasted and acid-etched) Type of abutment: MD Type of prosthesis: n.24 cement-retained fixed metal–ceramic Supported restoration: n. MD Position: MD Mean time after implant placement: MD	Treatment: NSMD + Diode Laser (Denlase 810/7, Beijing, China) Removal of the prosthesis: n.24 yes Session: n.1 Type of sample(s): submucosal Method(s) of sampling collection: sterile paper points Timing of collection after treatment: 1 month Microorganism identification technique: PCR Target: DNA	*Campylobacter gracilis*		Control group: n.5 Mean age: N/D Gender ratio: N/D Implant: n.48—with peri-implantitis: n.24 Implant design and type: n.24 tapered root form design with rough surface (sandblasted and acid-etched) Type of abutment: MD Type of prosthesis: n.24 cement-retained fixed metal–ceramic Supported restoration: n.MD Position: MD Mean time after implant placement: MD	Treatment: NSMD Removal of the prosthesis: n.24 yes Session: n.1 Type of sample(s): submucosal Method(s) of sampling collection: sterile paper points Timing of collection after treatment: 1 month Microorganism identification technique: PCR Target: DNA	*Campylobacter gracilis*	
			n.22	At 1 month: n.22			n.19	At 1 month: n.16
			*Campylobacter rectus*				*Campylobacter rectus*	
			n.20	At 1 month: n.20			n.20	At 1 month: n.18
			*Eubacterium nodatum*				*Eubacterium nodatum*	
			n.0	At 1 month: n.3			n.0	At 1 month
			*Fusobacterium nucleatum*				*Fusobacterium nucleatum*	
			n.24	At 1 month: n.24			n.24	At 1 month: n.24
			*Peptostreptococcus micros*				*Peptostreptococcus micros*	
			n.24	At 1 month: n.19			n.20	At 1 month: n.15
			*Porphyromonas gingivalis*				*Porphyromonas gingivalis*	
			n.19	At 1 month: n.12			n.18	At 1 month: n.18
			*Prevotella intermedia*				*Prevotella intermedia*	
			n.20	At 1 month: n.20			n.20	At 1 month: n.22
			*Prevotella nigrescens*				*Prevotella nigrescens*	
			n.23	At 1 month: n.22			n.19	At 1 month: n.16
			*Streptococcus costellatus*				*Streptococcus costellatus*	
			n.23	At 1 month: n.20			n. 23	At 1 month: n.20
			*Tannerella forsythia*				*Tannerella forsythia*	
			n.22	At 1 month: n.20			n.22	At 1 month: n.22
			*Treponema denticola*				*Treponema denticola*	
			n.24	At 1 month: n.22			n. 24	At 1 month: n.24
Bassetti M., 2014 *Clin Oral Implants Res* [39] RCT High risk Bredent Medical GmbH & Co. KG, Geschäftsbereich HELBO, Walldorf, Germany	Test group: n.20 (n.19 at 9 and 12 months) Mean age: 59 y.o.; range 27–78 y.o. Gender ratio: 10M/10F Implant: n.70—with peri-implantitis: n.20 Implant design and type: Straumann^®^ Dental Implant System with rough surface (sandblasted and acid-etched) Type of abutment: MD Type of prosthesis: MD Supported restoration: n.MD Position: tissue-level Mean time after implant placement: 7.3 years	Treatment: NSMD + glycine-based powder air-polishing (Air-Flow Master^®^, PerioPowder^®^, Perio-Flow^®^ nozzle) + aPDT (HELBO^®^ Photodynamic Systems GmbH) + Diode laser (HELBO TheraLite Laser, HELBO^®^ 3D Pocket Probe, Photodynamic Systems GmbH) Removal of the prosthesis: MD Session: n.6 (at baseline, after 1 week, 3, 6, 9 and 12 months) Type of sample(s): submucosal Method(s) of sampling collection: sterile paper points Timing of collection after treatment: 3, 6 and 12 months Microorganism identification technique: RT-PCR Target: DNA	*A. actinomycetemcomitans*		Control group: n.20 Mean age: 57 y.o.; range 29–75 y.o. Gender ratio: 10M/10F Implant: n.37—with peri-implantitis: n.20 Implant design and type: Straumann^®^ Dental Implant System with rough surface (sandblasted and acid-etched) Type of abutment: MD Type of prosthesis: MD Supported restoration: n.MD Position: tissue-level Mean time after implant placement: 7.2 years	Treatment: NSMD + glycine-based powder air-polishing (Air-Flow Master^®^, PerioPowder^®^, Perio-Flow^®^ nozzle) + Local minocycline hydrochloride microspheres (1 mg of Arestin^®^) Removal of the prosthesis: MD Session: n.6 (at baseline, after 1 week, 3, 6, 9 and 12 months) Type of sample(s): submucosal Method(s) of sampling collection: sterile paper points Timing of collection after treatment: 3, 6 and 12 months Microorganism identification technique: RT-PCR Target: DNA	*A. actinomycetemcomitans*	
			n.7 (35%) ≥10^5:^ n.1 (5%)	At 3 months n.6 (30%) ≥10^5:^ n.0 (0%)			n.7 (35%) ≥10^5^: n.2 (10%)	At 3 months n.8 (40%) ≥10^5^: n.0 (0%)
				At 6 months n.3 (15%) ≥10^5:^ n.0 (0%)				At 6 months n.5 (25%) ≥10^5^: n.0 (0%)
				At 12 months n.6 (32%) ≥10^5^: n.0 (0%)				At 12 months n.7 (35%) ≥10^5:^ n.0 (0%)
			*Campylobacter rectus*				*Campylobacter rectus*	
			n.6 ≥10^5^: n.3 (15%)	At 3 months: n.4 ≥10^5^: n.1 (5%)			n.17 ≥10^5^: n.3 (15%)	At 3 months: n.5 ≥10^5^: n.1 (5%) ^‡^
				At 6 months: n.3 ≥10^5^: n.1 (5%)				At 6 months: n.7 ≥10^5^: n.0 (0%) ^‡^
				At 12 months: n.8 ≥10^5^: n.2 (11%)				At 12 months: n.7 ≥10^5^: n.0 (0%) ^‡^
			*Capnocytophaga gingivalis*				*Capnocytophaga gingivalis*	
			n.20 ≥10^5:^ n.1 (5%)	At 3 months: n.20 ≥10^5:^ n.1 (5%)			n.20 ≥10^5^: n.5	At 3 months: n.20 ≥10^5:^ n.1 (5%)
				At 6 months: n.20 ≥10^5:^ n.2 (10%)				At 6 months: n.6 ≥10^5:^ n.1 (5%)
				At 12 months: n.19 ≥10^5:^ n.2 (11%)				At 12 months: n.20 ≥10^5:^ n.2 (10%)
			*Eikenella corrodens*				*Eikenella corrodens*	
			n.9 ≥10^5:^ n.4 (20%)	At 3 months: n.5 ≥10^5:^ n.1 (5%) ^‡^			n.13 ≥10^5:^ n.7 (35%)	At 3 months: n.5 ≥10^5:^ n.1 (5%) ^‡^
				At 6 months: n.6 ≥10^5:^ n.1 (5%)				At 6 months: n.2 ≥10^5:^ n.0 (0%) ^‡^
				At 12 months: n.6 ≥10^5:^ n.2 (11%)				At 12 months: n.8 ≥10^5:^ n.1 (5%) ^‡^
			*Eubacterium nodatum*				*Eubacterium nodatum*	
			n.11 ≥10^5:^ n.0 (0%)	At 3 months: n.9 ≥10^5:^ n.0 (0%)			n.11 ≥10^5:^ n.3 (15%)	At 3 months: n.8 ≥10^5:^ n.0 (0%)
				At 6, 12 months: n.12 ≥10^5^: n.0 (0%)				At 6, 12 months: n.9 ≥10^5^: n.0 (0%) *
			*Fusobacterium nucleatum*				*Fusobacterium nucleatum*	
			n.19 ≥10^5^: n.9 (45%)	At 3 months: n.12 ≥10^5^: n.3 (15%)			n.19 ≥10^5^: n.12 (60%)	At 3 months: n.14 ≥10^5^: n.3 (15%) ^‡^
				At 6 months: n.16 ≥10^5^: n.3 (15%) *				At 6 months: n.17 ≥10^5^: n.3 (15%) ^‡^
				At 12 months: n.14 ≥10^5^: n.2 (11%) *				At 12 months: n.15 ≥10^5^: n.3 (15%) ^‡^
			*Parvimonas micra*				*Parvimonas micra*	
			n.13 ≥10^5^: n.3 (15%)	At 3 months: n.13 ≥10^5^: n.1 (5%)			n.14 ≥10^5^: n.5 (25%)	At 3 months: n.11 ≥10^5^: n.3 (15%)
				At 6 months: n.11 ≥10^5^: n.1 (5%)				At 6 months: n.11 ≥10^5^: n.2 (10%)
				At 12 months: n.14 ≥10^5^: n.0 (0%)				At 12 months: n.16 ≥10^5^: n.2 (10%)
			*Porphyromonas gingivalis*				*Porphyromonas gingivalis*	
			n.5 ≥10^5^: n.2 (10%)	At 3 months: n.5 ≥10^5^: n.0 (0%) *			n.10 ≥10^5^: n.5 (25%)	At 3 months: n.9 ≥10^5^: n.1 (5%) *
				At 6 months: n.6 ≥10^5^: n.0 (0%) *				At 6 months: n.4 ≥10^5^: n.1 (5%) *
				At 12 months: n.4 ≥10^5^: n.0 (0%)				At 12 months: n.4 ≥10^5^: n.1 (5%) *
			*Prevotella intermedia*				*Prevotella intermedia*	
			n.6 ≥10^5^: n.2 (10%)	At 3 months: n.5 ≥10^5^: n.1 (5%)			n.6 ≥10^5^: n.0 (0%)	At 3 months: n.3 ≥10^5^: n.0 (0%) *
				At 6 months: n.5 ≥10^5^: n.0 (0%)				At 6 months: n.4 ≥10^5^: n.0 (0%)
				At 12 months: n.6 ≥10^5^: n.2 (11%)				At 12 months: n.4 ≥10^5^: n.0 (0%)
			*Tannerella forsythia*				*Tannerella forsythia*	
			n.11 ≥10^5^: n.4 (20%)	At 3 months: n.4 ≥10^5^: n.0 (0%) ^‡^			n.13 ≥10^5^: n.6 (30%)	At 3 months: n.5 ≥10^5^: n.1 (5%) ^‡^
				At 6 months: n.6 ≥10^5^: n.1 (5%) ^‡^				At 6 months: n.6 ≥10^5^: n.1 (5%) ^‡^
				At 12 months: n.7 ≥10^5^: n.2 (11%)				At 12 months: n.8 ≥10^5^: n.2 (10%) ^‡^
			*Treponema denticola*				*Treponema denticola*	
			n.8 ≥10^5^: n.2 (10%)	At 3 months: n.3 ≥10^5^: n.0 (0%) *			n.10 ≥10^5^: n.3 (15%)	At 3 months: n.3 ≥10^5^: n.0 (0%) ^‡^
				At 6 months: n.4 ≥10^5^: n.0 (0%)				At 6 months: n.4 ≥10^5^: n.1 (5%) ^‡^
				At 12 months: n.3 ≥10^5^: n.1 (5%)				At 12 months: n.4 ≥10^5^: n.1 (5%) *
Birang E., 2017 *J Laser Med Sci* [47] RCT Unclear risk No Funding	Test group: n.10 Mean age: N/D Gender ratio: N/D Implant: n.MD—with peri-implantitis: n.20 Implant design and type: MD Type of abutment: MD Type of prosthesis: MD Supported restoration: n.MD Position: MD Mean time after implant placement: MD	Treatment: NSMD + air polishing (Prophy-Jet) + diode laser + aPDT Removal of the prosthesis: MD Session: n.2 Type of sample(s): submucosal Method(s) of sampling collection: paper points Timing of collection after treatment: 3 months Microorganism identification technique: RT-PCR Target: MD	*A.actinomycetemcomitans*(log CFU/mL)		Control group: n.10 Mean age: N/D Gender ratio: N/D Implant: n.MD—with peri-implantitis: n.20 Implant design and type: MD Type of abutment: MD Type of prosthesis: MD Supported restoration: n.MD Position: MD Mean time after implant placement: MD	Treatment: NSMD + air polishing (Prophy-Jet) + diode laser Removal of the prosthesis: MD Session: n.2 Type of sample(s): submucosal Method(s) of sampling collection: paper points Timing of collection after treatment: 3 months Microorganism identification technique: RT-PCR Target: MD	*A. actinomycetemcomitans* (log CFU/mL)	
			0.91 ± 0.80	At 3 months 0.47 ± 0.64			1.12 ± 0.86	At 3 months 0.61 ± 0.62
			*Porphyromonas gingivalis* (log CFU/mL)				*Poprhyromonas gingivalis* (log CFU/mL)	
			1.42 ± 1.49	At 3 months 0.70 ± 0.99			1.68 ± 1.50	At 3 months 1.03 ± 1.44
			*Prevotella intermedia* (log CFU/mL)				*Prevotella intermedia* (log CFU/mL)	
			1.04 ± 1.30	At 3 months 0.39 ± 0.58			1.27 ± 1.11	At 3 months 0.65 ± 1.19
			*Treponema denticola* (log CFU/mL)				*Treponema denticola* (log CFU/mL)	
			0.53 ± 0.63	At 3 months 0.21 ± 0.46			0.48 ± 0.55	At 3 months 0.28 ± 0.44
			*Tannerella forsythia* (log CFU/mL)				*Tannerella forsythia* (log CFU/mL)	
			0.43 ± 0.55	At 3 months 0.14 ± 0.24			0.31 ± 0.55	At 3 months 0.15 ± 0.27
Bombeccari, G.P., 2013 *Implant Dent* [40] RCT High risk No Funding	Test group: n.20 Mean age: N/D Gender ratio: N/D Implant: n. MD—with peri-implantitis: n.20 Implant design and type: Nobel Biocare^®^ with rough surfaces Type of abutment: MD Type of prosthesis: MD Supported restoration: n. MD Position: MD Mean time after implant placement: MD	Treatment: SMD + local 0.2% CHX + aPDT + Diode laser + 0.2% CHX (8 h/2 weeks) Removal of the prosthesis: no Session: n.1 Type of sample(s): N/D Method(s) of sampling collection: paper strips Timing of collection after treatment: after treatment, 3, 6 months Microorganism identification technique: bacterial cultures Target: MD	*Porphyromans gingivalis* (log CFU/mL)		Control group: n.20 Mean age: N/D Gender ratio: N/D Implant: n. MD—with peri-implantitis: n.20 Implant design and type: Nobel Biocare^®^ with a rough surface Type of abutment: MD Type of prosthesis: MD Supported restoration: n. MD Position: MD Mean time after implant placement: MD	Treatment: SMD + local 0.2% CHX (8 h for 2 weeks) Removal of the prosthesis: no Session: n.1 Type of sample(s): N/D Method(s) of sampling collection: paper strips Timing of collection after treatment: after treatment, 3, 6 months Microorganism identification technique: bacterial cultures Target: MD	*Porphyromonas gingivalis* (log CFU/mL)	
			1.93	After treatment 0.44 ± 0.14			1.93	After treatment 1.05 ± 0.02
			*Prevotella intermedia* (log CFU/mL)				*Prevotella intermedia* (log CFU/mL)	
			1.93	After treatment 0.57 ± 0.34			1.95	After treatment 1.18 ± 0.23
			*A. actinomycetemcomitans* (log CFU/mL)				*A. actinomycetemcomitans* (log CFU/mL)	
			1.79	After treatment 0.45 ± 0.04			1.81	After treatment 1.04 ± 0.12 ^‡^
			*Total Anaerobic Bacteria* (log CFU/mL)				*Total Anaerobic Bacteria* (log CFU/mL)	
			2.35 ± 0.02	After treatment 0.98 ± 0.20			2.37 ± 0.03	After treatment 1.58 ± 0.34
				At 3 months 1.50				At 3 months 1.86
				At 6 months 1.77				At 6 months 2.06
Cha, J.K., 2019 *J Dent Res* [41] RCT High risk Sunstar Inc. and Weimer Pharma	Test group: n.25 Mean age: 60.2 y.o.; range 40–83 y.o. Gender ratio: 15M/10F Implant: n.N/D—with peri-implantitis: n.25 Implant design and type: nonmodified turned surface n.1; TiOblast n.1; OsseoTite n.4; sandblasted and acid-etched n.11; resorbable blast media n.2 Type of abutment: MD Type of prosthesis: MD Supported restoration: n.MD Position: MD Mean time after implant placement: MD	Treatment: SMD + powder air-polishing (Air-Flow Master^®^) + Local minocycline ointment + Systemic AMX (500 mg 3/3 d) + ibuprofen (600 mg 3/3 d) Removal of the prosthesis: MD Session: n.4 (1 week, 1.3 months: NSMD + Local minocycline 1 mg) Type of sample(s): MD Method(s) of sampling collection: sterile paper points Timing of collection after treatment: 3 and 6 months Microorganism identification technique: RT-PCR Target: MD	*Campylobacter rectus* (%)		Control group: n.25 Mean age: 63.0 y.o.; range 46–84 y.o. Gender ratio: 10M/15F Implant: n.N/D—with peri-implantitis: n.25 Implant design and type: TiUnite n.3; OsseoSpeed n.2; OsseoTite n.2; sandblasted and acid-etched n.15; Type of abutment: MD Type of prosthesis: MD Supported restoration: n.MD Position: MD Mean time after implant placement: MD	Treatment: SMD + powder air-polishing (Air-Flow Master^®^) + placebo ointment + Systemic AMX (500 mg 3/3 d) + ibuprofen (600 mg 3/3 d) Removal of the prosthesis: MD Session: n.4 (1 week, 1, 3 months: NSMD + placebo) Type of sample(s): MD Method(s) of sampling collection: sterile paper points Timing of collection after treatment: 3 and 6 months Microorganism identification technique: RT-PCR Target: MD	*Campylobacter rectus* (%)	
			>90.0	At 3 months: >60.0			>80.0	At 3 months: >70.0
				At 6 months: >40.0				At 6 months: >50.0
			*Eubacterium nodatum* (%)				*Eubacterium nodatum* (%)	
			<10.0	At 3 months: <10.0			<20.0	At 3 months: 0.0
				At 6 months: 0.0				At 6 months: >0.0
			*Fusobacterium nucleatum* (%)				*Fusobacterium nucleatum* (%)	
			100	At 3.6 months: 100			100	At 3.6 months: 100
			*Peptostreptococcus micros* (%)				*Peptostreptococcus micros* (%)	
			80.0	At 3.6 months: 50			>80.0	At 3.6 months: 60
			*Porphyromonas gingivalis* (%)				*Porphyromonas gingivalis* (%)	
			>30.0	At 3.6 months: 0			>30.0	At 3.6 months: >0
			*Prevotella intermedia* (%)				*Prevotella intermedia* (%)	
			>50.0	At 3 months: >10.0			>60.0	At 3 months: >40.0
				At 6 months: 30.0				At 6 months: >40.0
			*Prevotella nigrescens* (%)				*Prevotella nigrescens* (%)	
			>70.0	At 3 months: >40.0			>60.0	At 3 months: 50.0
				At 6 months: >40.0				At 6 months: 40.0
			*Tannerella fortsythia* (%)				*Tannerella forsythia* (%)	
			>60.0	At 3 months: >20.0			>70.0	At 3 months: >50.0
				At 6 months: 0.00				At 6 months: >10.0
			*Treponema denticola* (%)				*Treponema denticola* (%)	
			>40.0	At 3 months: >0.00			>30.0	At 3 months: >20.0
				At 6 months: >10.0				At 6 months: >20.0
			*Total Orange-Complex Bacteria* (%)				*Total Orange-Complex Bacteria* (%)	
			100	At 3.6 months: 100			100	At 3.6 months: 100
			*Total Red-Complex Bacteria* (%)				*Total Red-Complex Bacteria* (%)	
			87.5	At 3 months: 25.0			81.8	At 3 months: 59.1
				At 6 months: 12.5				At 6 months: 31.8
Chen, J.H., 2022 *Laser Med Sci* [42] RCT Unclear risk Southern Taiwan Science Park	Test group: n.11 Mean age: MD Gender ratio: MD Implant: n.MD—with peri-implantitis: n.13 Implant design and type: MD Type of abutment: MD Type of prosthesis: MD Supported restoration: n.MD Position: MD Mean time after implant placement: MD	Treatment: Er:YAG Removal of the prosthesis: MD Session: n.3 at baseline, at 2 and 4 weeks Type of sample(s): submucosal Method(s) of sampling collection: paper points Timing of collection after treatment: 3 and 6 months Microorganism identification technique: MD Target: MD	*Total Anaerobic Bacteria* (log CFU/mL)		Control group: n.12 Mean age: MD Gender ratio: MD Implant: n.MD—with peri-implantitis: n.12 Implant design and type: MD Type of abutment: MD Type of prosthesis: MD Supported restoration: n.MD Position: MD Mean time after implant placement: MD	Treatment: NSMD Removal of the prosthesis: MD Session: n.1 Type of sample(s): submucosal Method(s) of sampling collection: paper points Timing of collection after treatment: 3 and 6 months Microorganism identification technique: MD Target: MD	*Total Anaerobic Bacteria* (log CFU/mL)	
			9.23 ± 3.06	At 3 months 9.43 ± 1.85			12.02 ± 1.90	At 3 months 9.05 ± 2.74 *
				At 6 months 8.80 ± 2.49				At 6 months 8.66. ± 2.55 *
Galofré, M., 2018 *J Periodontal Res* [43] RCT Unclear risk Sunstar Suisse and BioGaia	Test group: n.11 Mean age: 61.7 ± 7.0 Gender ratio: 8M/3F Implant: n.M Implant with peri-implantitis: n.11 Implant design and type: MD Type of abutment: MD Type of prosthesis: MD Supported restoration: single crown (n.36); fixed partial prosthesis (n.64) Position: MD Mean time after implant placement: MD	Treatment: NSMD+ *Lactobacillus reuteri (*Prodentis, PerioBalance^®^, 1 lozenge for 30 d) Removal of the prosthesis: MD Session: n.1 Type of sample(s): submucosal Method(s) of sampling collection: sterile paper points Timing of collection after treatment: 1 and 3 months Microorganism identification technique: RT-PCR Target: MD	*A. actinomycetemcomitans* (log CFU/mL)		Control group: n.11 Mean age: 56.8 ± 9.3 Gender ratio: 5M/6F Implant: n.MD Implant with peri-implantitis: n.11 Implant design and type: MD Type of abutment: MD Type of prosthesis: MD Supported restoration: single crown (n.36); fixed partial prosthesis (n.64) Position: MD Mean time after implant placement: MD	Treatment: NSMD + Placebo (1 lozenges for 30 d) Removal of the prosthesis: MD Session: n.1 Type of sample(s): submucosal Method(s) of sampling collection: sterile paper points Timing of collection after treatment: 1 and 3 months Microorganism identification technique: RT-PCR Target: MD	*A. actinomycetemcomitans* (log CFU/mL)	
			0.00 ± 0.00	At 1 and 3 month 0.00 ± 0.00			0.00 ± 0.00	At 1 and 3 month 0.00 ± 0.00
			*Campylobacter rectus* (log CFU/mL)				*Campylobacter rectus* (log CFU/mL)	
			5.97 ± 1.16	At 1 month 4.95 ± 2.58			6.07 ± 0.86	At 1 month 5.67 ± 1.98
				At 3 months 5.80 ± 1.02				At 3 months 6.20 ± 0.87
			*Eikenella corrodens* (log CFU/mL)				*Eikenella corrodens* (log CFU/mL)	
			4.36 ± 2.94	At 1 month 4.48 ± 2.99			5.72 ± 1.12	At 1 month 5.00 ± 1.88
				At 3 months 3.77 ± 2.66				At 3 months 4.96 ± 1.79
			*Fusobacterium nucleatum* (log CFU/mL)				*Fusobacterium nucleatum* (log CFU/mL)	
			6.78 ± 0.97	At 1 month 5.60 ± 2.92			6.81 ± 0.66	At 1 month 6.59 ± 0.72
				At 3 months 6.64 ± 1.18				At 3 months 6.94 ± 0.50
			*Peptostreptococcus micros* (log CFU/mL)				*Peptostreptococcus micros* (log CFU/mL)	
			5.88 ± 0.78	At 1 month 4.81 ± 2.48			6.10 ± 0.61	At 1 month 5.30 ± 1.94
				At 3 months 5.32 ± 1.94				At 3 months 5.97 ± 0.69
			*Porphyromonas gingivalis* (log CFU/mL)				*Porphyromonas gingivalis* (log CFU/mL)	
			5.20 ± 2.90	At 1 month 5.74 ± 3.08			4.81 ± 3.29	At 1 month 4.75 ± 3.34
				At 3 months 5.21 ± 2.86				At 3 months 4.91 ± 3.43
			*Prevotella intermedia* (log CFU/mL)				*Prevotella intermedia* (log CFU/mL)	
			6.10 ± 2.34	At 1 month 7.18 ± 0.88			6.43 ± 2.22	At 1 month 5.67 ± 2.96
				At 3 months 6.06 ± 2.18				At 3 months 5.47 ± 2.91
			*Tannerella forsythia* (log CFU/mL)				*Tannerella forsythia* (log CFU/mL)	
			5.46 ± 1.20	At 1 month 5.60 ± 1.09			5.06 ± 1.87	At 1 month 4.54 ± 2.34
				At 3 months 4.78 ± 2.45				At 3 months 4.89 ± 2.48
			*Treponema denticola* (log CFU/mL)				*Treponema denticola*(log CFU/mL)	
			3.80 ± 3.16	At 1 month 4.04 ± 3.26			4.33 ± 2.92	At 1 month 3.73 ± 3.12
				At 3 months 3.14 ± 3.14				At 3 months 3.30 ± 3.26
			*Total Bacteria Counts* (log CFU/mL)				*Total Bacteria Counts* (log CFU/mL)	
			9.05 ± 1.11	At 1 month 9.46 ± 0.93			9.31 ± 0.67	At 1 month 9.26 ± 0.66
				At 3 months 8.96 ± 1.10				At 3 months 9.33 ± 0.74
Laleman, I., 2020 *Clin Oral Implants Res* [44] RCT High risk BioGaia AB and Acteon	Test group: n.9 Mean age: 64 ± 11 Gender ratio: 5M/4F Implant: n.MD Implant with peri-implantitis: n.9 Implant design and type: N/D Type of abutment: N/D Type of prosthesis: N/D Supported restoration: n.N/D Position: N/D Mean time after implant placement: N/D	Treatment: NSMD + powder air-polishing (Air-N-Go Easy, Acteon) + Probiotic (*Lactobaillus reuteri,* BioGaia AB) Removal of the prosthesis: MD Session: n.1 Type of sample(s): submucosal, tongue and saliva Method(s) of sampling collection: sterile paper points; sterile cotton swab Timing of collection after treatment: 2, 4 and 8 months Microorganism identification technique: RT-PCR Target: DNA, 16s rRNA	Submucosal/Saliva/Tongue (log CFU/mL):		Control group: n.10 Mean age: 69 ± 9 Gender ratio: 4M/6F Implant: n.MD Implant with peri-implantitis: n.10 Implant design and type: N/D Type of abutment: N/D Type of prosthesis: N/D Supported restoration: n.N/D Position: N/D Mean time after implant placement: N/D	Treatment: NSMD + powder air-polishing (Air-N-Go Easy) + Placebo Removal of the prosthesis: MD Session: n.1 Type of sample(s): submucosal, tongue and saliva Method(s) of sampling collection: sterile paper points; sterile cotton swab Timing of collection after treatment: 2, 4 and 8 months Microorganism identification technique: RT-PCR Target: DNA, 16s rRNA	Submucosal/Saliva/Tongue (log CFU/mL):	
			*A. actinomytemcomitans*				*A. actinomytemcomitans*	
			3.09 ± 2.54/ 3.61 ± 2.27/ 3.56 ± 2.26	At 6 weeks 3.71 ± 1.66/3.52 ± 2.71/3.50 ± 2.11			3.74 ± 2.47/ 3.24 ± 1.87/ 2.76 ± 2.10	At 6 weeks 3.67 ± 2.30/2.67 ± 2.45/2.78 ± 2.09
				At 3 months 3.62 ± 2.43/3.83 ± 1.78/2.80 ± 2.26 *				At 3 months 3.43 ± 2.33/2.71 ± 2.07 */2.53 ± 1.83/
				At 6 months 2.44 ± 2.41/3.37 ± 2.19/2.42 ± 2.44 *				At 6 months 2.45 ± 2.92/2.36 ± 2.14 */2.88 ± 2.06
			*Fusobacterium nucleatum*				*Fusobacterium nucleatum*	
			6.93 ± 0.78/ 6.17 ± 0.61/ 6.14 ± 1.55	At 6 weeks 6.72 ± 1.29/6.09 ± 1.08/6.31 ± 1.34			6.87 ± 0.90/ 6.18 ± 0.51/ 6.54 ± 1.11	At 6 weeks 6.69 ± 0.94/6.11 ± 0.95/6.67 ± 1.12
				At 3 months 6.84 ± 1.21/6.35 ± 1.20/6.48 ± 1.31				At 3 months 6.87 ± 1.21/6.31 ± 0.59/6.75 ± 0.82
				At 6 months 6.68 ± 1.23/6.43 ± 1.08/6.63 ± 1.23 *				At 6 months 6.90 ± 1.25/6.34 ± 0.65/6.63 ± 1.22
			*Porphyromonas gingivalis*				*Porphyromonas gingivalis*	
			5.13 ± 3.14/ 5.12 ± 2.09/ 3.72 ± 2.18	At 6 weeks 5.27 ± 3.10/4.58 ± 2.65/3.38 ± 1.98 *			3.51 ± 3.37/ 2.79 ± 2.98/ 2.61 ± 2.32	At 6 weeks 3.49 ± 3.33/3.27 ± 2.91/2.45 ± 2.21
				At 3 months 5.22 ± 3.16/4.78 ± 2.74/3.45 ± 2.05 *				At 3 months 3.08 ± 3.48/2.93 ± 2.83/1.60 ± 2.17 *
				At 6 months 5.21 ± 3.13/4.91 ± 2.80/3.54 ± 2.07 *				At 6 months 3.10 ± 3.48/2.79 ± 3.08/2.25 ± 2.45
			*Prevotella intermedia*				*Prevotella intermedia*	
			2.46 ± 1.97/ 1.72 ± 2.07/ 1.13 ± 1.71	At 6 weeks 2.41 ± 2.44/1.39 ± 2.15/0.39 ± 1.17 *			2.04 ± 2.28/ 1.89 ± 2.43/ 1.92 ± 2.50	At 6 weeks 1.35 ± 2.26/1.73 ± 2.38/1.81 ± 2.35
				At 3 months 1.53 ± 2.39 */1.00 ± 1.99/0.44 ± 1.32 *				At 3 months 1.40 ± 2.32/1.49 ± 2.40/1.42 ± 2.33
				At 6 months 1.06 ± 2.11 */1.59 ± 2.41/0.44 ± 1.31 *				At 6 months 2.02 ± 2.19/1.45 ± 2.34/1.44 ± 2.33
Passariello, C., 2012 *Eur J Inflamm* [45] RCT High risk No Funding	Test group: n.64 Mean age: 37.1 ± 6.8 y.o.; range 21–53 y.o. Gender ratio: 35M/29F Implant: n.MD Implant with peri-implantitis: n.64 Implant design and type: MD Type of abutment: MD Type of prosthesis: MD Supported restoration: n.MD Position: MD Mean time after implant placement: MD	Treatment: SMD + Serratiopeptidase (5 mg/12 h/15 d) + Systemic AMX-clavulanic acid (2 g/d) or clindamycin (1.2 g/d) Removal of the prosthesis: yes Session: n.1 Type of sample(s): submucosal Method(s) of sampling collection: paper points Timing of collection after treatment: 6 months Microorganism identification technique: RT-PCR Target: DNA, 16s rRNA	*Pseudomonas aeruginosa* (%)		Control group: n.64 Mean age: 36.6 ± 6.6 y.o.; range 21–50 y.o. Gender ratio: 32M/32F Implant: n.MD Implant with peri-implantitis: n.64 Implant design and type: MD Type of abutment: MD Type of prosthesis: MD Supported restoration: n.MD Position: MD Mean time after implant placement: MD	Treatment: SMD + Systemic AMX-clavulanic acid (2 g/d) or clindamycin (1.2 g/d) Removal of the prosthesis: yes Session: n.1 Type of sample(s): submucosal Method(s) of sampling collection: paper points Timing of collection after treatment: 6 months Microorganism identification technique: RT-PCR Target: DNA and 16s rRNA	*Pseudomonas aeruginosa* (%)	
			n.12 (18.75%)	At 6 months n.0 (0%)			n.13 (20.3%)	At 6 months n.0 (0%)
			*Staphylococcus aureus* (%)				*Staphylococcus aureus* (%)	
			n.13 (20.3%)	At 6 months n.2 (1.28%)			n.11 (17.2%)	At 6 months n.3 (1.92%)
Shibli, J.A., 2019 *Braz Oral Res* [46] RCT Unclear risk São Paulo Research Foundation	Test group: n.20 Mean age: N/D Gender ratio: N/D Implant: n.MD Implant with peri-implantitis: n.20 Implant design and type: machined surface with external hexagon Type of abutment: MD Type of prosthesis: MD Supported restoration: n.MD Position: MD Mean time after implant placement: N/D	Treatment: NSMD + Systemic MTZ (400 mg 3/d for 14 d) + AMZ (500 mg 3/d for 14 d) Removal of the prosthesis: MD Session: n.5 (NSMD at 3, 6, 9 and 12 months) Type of sample(s): submucosal Method(s) of sampling collection: curette Timing of collection after treatment: 14 d, 3, 6, 9 and 12 months Microorganism identification technique: MD Target: DNA	*Total Red-Complex Bacteria* (%)		Control group: n.20 Mean age: N/D Gender ratio: N/D Implant: n.MD Implant with peri-implantitis: n.20 Implant design and type: machined surface with external hexagon Type of abutment: MD Type of prosthesis: MD Supported restoration: n.MD Position: MD Mean time after implant placement: N/D	Treatment: NSMD + Placebo Removal of the prosthesis: MD Session: n.5 (NSMD at 3, 6, 9 and 12 months) Type of sample(s): submucosal Method(s) of sampling collection: curette Timing of collection after treatment: 14 d, 3, 6, 9 and 12 months Microorganism identification technique: MD Target: DNA	*Total Red-Complex Bacteria* (%)	
			>32.7	At 14 days 3.5			24.7	At 14 days 5.5
				At 3 months 5.5				At 3 months 8.0
				At 6 months 14.7				At 6 months 10.9
				At 12 months 15.0 *				At 12 months 18.6
			*Total Orange-Complex Bacteria* (%)				*Total Orange-Complex Bacteria* (%)	
			45.3	At 14 days 22.9			51.3	At 14 days 37.0
				At 3 months 42.4				At 3 months 30.7
				At 6 months 42.2				At 6 months 30.1
				At 12 months 45.6				At 12 months 37.7 *

Abbreviations: number, “n”; male, “M”; female, “F”; missing data, “MD”; milligram, “mg”; milliliters, “mL”; millimeters, “mm”; picograms, “pg”; nanograms, “ng”; pro re nata, “prn”; percentages, “%”; logarithm, “log”; Colony Forming Unit, “CFU”; day, “d”; minutes, “min”; Randomized Controlled Trial, “RCT”; non-surgical mechanical debridement, “NSMD”; surgical mechanical debridement, “SMD”; erbium-doped yttrium aluminium garnet, “Er:YAG”; Aggregatibacter, “*A.*”; amoxicillin, “AMX”; metronidazole, “MTZ”; chlorhexidine, “CHX”; cetylpyridinium chloride, “CPC”; polymerase chain reaction, “PCR”; real time PCR, “RT-PCR”; DeoxyriboNucleic Acid, “DNA”; ribosomial RiboNucleic Acid, “rRNA”; Plaque Index, “PI”; modified Plaque Index, “mPII”; Full Mouth Plaque Score, “FMPS”; Gingival Index, “GI”; Probing Depth, “PD”; Clinical Attachment Level, “CAL”; Bleeding on Probing, “BoP”; modified Sulcus Bleeding Index, “mSBI”; Full Mouth Bleeding Score, “FMBS”; inflammatory exudation, “IE”; Marginal Bone Level, “MBL”; titanium, “Ti”; metalloproteinase, “MMP”; interleukin, “IL”;Tumor Necrosis Factor, “TNF”; statistically significant difference from baseline, “*”; statistically significant difference between the test and control group, “^‡^”. The study population comprised 432 systemically healthy, non-smoking, partially edentulous subjects (215 subjects from the test groups of the studies and 217 controls), in whom a total of 492 peri-implantitis sites were treated (246 peri-implantitis-treated sites in both the test and control groups of the studies).

## Data Availability

The original contributions presented in the study are included in the article/Supplementary Material, further inquiries can be directed to the corresponding authors.

## Supplementary Materials

The following supporting information can be downloaded at: https://www.mdpi.com/article/10.3390/microorganisms12101965/s1, File S1: Bacteria counts (log CFU/mL) over time. File S2: Quality assessment of included studies.
