# Supplementary material for: Peri-Implantitis-Associated Microbiota before and after Peri-Implantitis Treatment, the Biofilm “Competitive Balancing” Effect: A Systematic Review of Randomized Controlled Trials
Source: Microorganisms. 2024 Sep 28;12(10):1965. doi: 10.3390/microorganisms12101965 (PMC11509653; doi:10.3390/microorganisms12101965)
Supplement: Supplementary file 1 [file microorganisms-12-01965-s001.zip › Supplementary File S1.pdf]

Microorganisms
Peri-Implantitis-Associated Microbiota before and after Peri-Implantitis Treatment, the Biofilm "Competitive Balancing" Effect: A Systematic Review of Randomized Controlled Trials

Federica Di Spirito [1,*], Massimo Pisano [1,†], Maria Pia Di Palo [1,†], Gianluigi Franci [1], Antonio Rupe [1], Antonino Fiorino [2,‡] and Carlo Rengo [1,*,‡]

Supplementary File S1 – Bacteria counts (log CFU/mL) over time

Table S1. *Porphyromonas gingivalis* counts (log CFU/mL) of test and control group, extracted from the studies included in the present systematic review; number of peri-implantitis sites treated (n.) and peri-implantitis treatment; weighted average counts at baseline, after treatment, one month, 6 weeks, 3 months, 6 months, and 12 months; mean absolute counts deviation from the baseline.

	Baseline	After treatment	1 month	6 weeks	3 months	6 months	12 months
Porphyromonas gingivalis	5.73 ± 1.12 (20) a.<br>5.29 ± 1.64 (20) b.<br>1.42 ± 1.49 (20) c.<br>1.68 ± 1.50 (20) d.<br>5.20 ± 2.90 (11) e.<br>4.81 ± 3.29 (11) f.<br>5.13 ± 3.14 (9) g.<br>3.72 ± 2.18 (9) g.<br>5.12 ± 2.09 (9) g.<br>3.51 ± 3.37 (10) h.<br>2.61 ± 2.32 (10) h.<br>2.79 ± 2.98 (10) h.<br>1.93 (20) i.<br>1.93 (20) j.	0.44 ± 0.14 (20) i.<br>1.05 ± 0.02 (20) j.	5.74 ± 3.08 (11) e.<br>4.75 ± 3.34 (11) f.	5.27 ± 3.10 (9) g.<br>3.38 ± 1.98 (9) g.<br>4.58 ± 2.65 (9) g.<br>3.49 ± 3.33 (10) h.<br>2.45 ± 2.21 (10) h.	5.21 ± 2.86 (11) e.<br>4.91 ± 3.43 (11) f.<br>0.70 ± 0.99 (20) c.<br>1.03 ± 1.44 (20) d.<br>5.22 ± 3.16 (9) g.<br>3.45 ± 2.05 (9) g.<br>4.78 ± 2.74 (9) g.<br>3.08 ± 3.48 (10) h.<br>1.60 ± 2.17 (10) h.<br>2.93 ± 2.83 (10) h.	3.24 ± 1.52 (20) a.<br>3.96 ± 1.11 (20) b.<br>5.21 ± 3.13 (9) g.<br>3.54 ± 2.07 (9) g.<br>4.91 ± 2.80 (9) g.<br>3.10 ± 3.48 (10) h.<br>2.25 ± 2.45 (10) h.<br>2.79 ± 3.08 (10) h.	4.67 ± 1.44 (20) a.<br>4.48 ± 1.35 (20) b.
X	3.55 ± 1.60	0.75 ± 0.08	5.24 ± 3.21	3.80 ± 2.66	2.90 ± 0.75	3.58 ± 0.74	4.58 ± 1.40
D		-2.80 ± 1.60	+1.69 ± 3.59	+0.25 ± 3.10	-0.65 ± 0.85	+0.03 ± 0.86	+1.03 ± 1.64

Abbreviations: weighted average, "X"; mean absolute deviation from the baseline, "*D*"; NSMD plus CHX plus local antibiotics plus diode laser plus aPDT, "a."; NSMD plus CHX plus local antibiotics plus diode laser, "b"; NSMD plus air-polishing plus diode laser plus aPDT, "c"; NSMD plus air-polishing plus diode laser, "d"; NSMD plus probiotics, "e"; NSMD, "f"; NSMD plus air-polishing plus probiotics, "g"; NSMD plus air-polishing, "h"; SMD plus air-polishing plus diode laser plus aPDT, "i"; SMD plus CHX, "j".

Table S2. *Tannerella forsythia* counts (log CFU/mL) of test and control group, extracted from the studies included in the present systematic review; number of peri-implantitis sites treated (n.) and peri-implantitis treatment; weighted average counts at baseline, one month, 3 months, 6 months, and 12 months; mean absolute counts deviation from the baseline.

	Baseline	1 month	3 months	6 months	12 months
Tannerella forsythia	4.22 ± 1.73 (20) a.<br>4.46 ± 1.21 (20) b.<br>0.43 ± 0.55 (20) c.<br>0.31 ± 0.55 (20) e.<br>5.46 ± 1.20 (11) d.<br>5.06 ± 1.87 (11) f.	5.60 ± 1.09 (11) e.<br>4.54 ± 2.34 (11) f.	4.78 ± 2.45 (11) e.<br>4.89 ± 2.48 (11) f.<br>0.14 ± 0.24 (20) c.<br>0.15 ± 0.27 (20) d.	2.64 ± 1.23 (20) a.<br>2.98 ± 1.18 (20) b.	3.33 ± 1.74 (20) a.<br>3.86 ± 1.89 (20) b.
X	2.98 ± 1.12	5.07 ± 1.72	1.79 ± 0.62	2.81 ± 0.85	3.60 ± 1.82
D		+2.09 ± 2.05	+1.19 ± 0.50	-0.17 ± 0.27	+0.62 ± 2.14

Abbreviations: weighted average, "X"; mean absolute deviation from the baseline, "*D*"; NSMD plus CHX plus local antibiotics plus diode laser plus aPDT, "a."; NSMD plus CHX plus local antibiotics plus diode laser, "b"; NSMD plus air-polishing plus diode laser plus aPDT, "c"; NSMD plus air-polishing plus diode laser, "d"; NSMD plus probiotics, "e"; NSMD, "f".

Table S3. *Treponema denticola* counts (log CFU/mL) of test and control group, extracted from the studies included in the present systematic review; number of peri-implantitis sites treated (n.) and peri-implantitis treatment; weighted average counts at baseline, one month, 3 months, 6 months, and 12 months; mean absolute counts deviation from the baseline.

	Baseline	1 month	3 months	6 months	12 months
Treponema denticola	4.19 ± 1.92 (20) a.<br>4.54 ± 1.08 (20) b.<br>0.53 ± 0.63 (20) c.<br>0.48 ± 0.55 (20) d.<br>3.80 ± 3.16 (11) e.<br>4.33 ± 2.92 (11) f.	4.04 ± 3.26 (11) e.<br>3.73 ± 3.12 (11) f.	0.21 ± 0.46 (20) c.<br>0.28 ± 0.44 (20) d.<br>3.14 ± 3.14 (11) e.<br>3.30 ± 3.26 (11) f.	3.12 ± 1.09 (20) a.<br>3.41 ± 0.89 (20) b.	3.75 ± 1.79 (20) a.<br>3.96 ± 1.88 (20) b.
X	2.78 ± 1.48	3.89 ± 3.19	1.20 ± 0.54	3.27 ± 0.69	3.86 ± 1.84
D		+1.11 ± 3.51	-1.58 ± 0.94	+0.49 ± 0.79	+1.08 ± 2.36

Abbreviations: weighted average, "X"; mean absolute deviation from the baseline, "*D*"; NSMD plus CHX plus local antibiotics plus diode laser plus aPDT, "a."; NSMD plus CHX plus local antibiotics plus diode laser, "b"; NSMD plus air-polishing plus diode laser plus aPDT, "c"; NSMD plus air-polishing plus diode laser, "d"; NSMD plus probiotics, "e"; NSMD, "f".

Table S4. *Campylobacter rectus* counts (log CFU/mL) of test and control group, extracted from the studies included in the present systematic review; number of peri-implantitis sites treated (n.) and peri-implantitis treatment; weighted average counts at baseline, one month, and 3 months; mean absolute counts deviation from the baseline.

	Baseline	1 month	3 months
Campylobacter rectus	5.97 ± 1.16 (11) e.<br>6.07 ± 0.86 (11) f.	4.95 ± 2.58 (11) e.<br>5.67 ± 1.98 (11) f.	5.80 ± 1.02 (11) e.<br>6.20 ± 0.87 (11) f.
X	6.02 ± 0.72	5.31 ± 1.63	6.00 ± 0.67
D		-0.71 ± 1.78	-0.02 ± 0.98

Abbreviations: weighted average, "X"; mean absolute deviation from the baseline, "*D*"; NSMD plus probiotics, "e"; NSMD, "f".

Table S5. *Fusobacterium nucleatum* counts (log CFU/mL) of test and control group, extracted from the studies included in the present systematic review; number of peri-implantitis sites treated (n.) and peri-implantitis treatment; weighted average counts at baseline, one month, 6 weeks, 3 months and 6 months; mean absolute counts deviation from the baseline.

	Baseline	1 month	6 weeks	3 months	6 months
Fusobacterium nucleatum	6.78 ± 0.97 (11) e.<br>6.81 ± 0.66 (11) f.<br>6.93 ± 0.78 (9) g.<br>6.14 ± 1.55 (9) g.<br>6.17 ± 0.61 (9) g.<br>6.87 ± 0.90 (10) h.<br>6.54 ± 1.11 (10) h.<br>6.18 ± 0.51 (10) h.	5.60 ± 2.92 (11) e.<br>6.59 ± 0.72 (11) f.	6.72 ± 1.29 (9) g.<br>6.31 ± 1.34 (9) g.<br>6.09 ± 1.08 (9) g.<br>6.69 ± 0.94 (10) h.<br>6.67 ± 1.12 (10) h.<br>6.11 ± 0.95 (10) h.	6.64 ± 1.18 (11) e.<br>6.94 ± 0.50 (11) f.<br>6.84 ± 1.21 (9) g.<br>6.48 ± 1.31 (9) g.<br>6.35 ± 1.20 (9) g.<br>6.87 ± 1.21 (10) h.<br>6.75 ± 0.82 (10) h.<br>6.31 ± 0.59 (10) h.	6.68 ± 1.23 (9) g.<br>6.63 ± 1.23 (9) g.<br>6.43 ± 1.08 (9) g.<br>6.90 ± 1.25 (10) h.<br>6.63 ± 1.22 (10) h.<br>6.34 ± 0.65 (10) h.
X	6.57 ± 1.07	6.10 ± 1.50	6.44 ± 0.72	6.66 ± 0.32	6.60 ± 0.62
D		-0.47 ± 1.84	-0.13 ± 0.77	+0.09 ± 1.12	+0.03 ± 1.24

Abbreviations: weighted average, "X"; mean absolute deviation from the baseline, "*D*"; NSMD plus probiotics, "e"; NSMD, "f"; NSMD plus air-polishing plus probiotics, "g"; NSMD plus air-polishing, "h".

Table S6. *Peptostreptococcus micros* counts (log CFU/mL) of test and control group, extracted from the studies included in the present systematic review; number of peri-implantitis sites treated (n.) and peri-implantitis treatment; weighted average counts at baseline, one month, and 3 months; mean absolute counts deviation from the baseline.

	Baseline	1 month	3 months
Peptostreptococcus micros	5.88 ± 0.78 (11) e.<br>6.10 ± 0.61 (11) f.	4.81 ± 2.48 (11) e.<br>5.30 ± 1.94 (11) f.	5.32 ± 1.94 (11) e.<br>5.97 ± 0.69 (11) f.
X	5.99 ± 0.70	5.05 ± 2.21	5.65 ± 1.32
D		-0.94 ± 2.32	-0.34 ± 1.49

Abbreviations: weighted average, "X"; mean absolute deviation from the baseline, "*D*"; NSMD plus probiotics, "e"; NSMD, "f".

Table S7. *Prevotella intermedia* counts (log CFU/mL) of test and control group, extracted from the studies included in the present systematic review; number of peri-implantitis sites treated (n.) and peri-implantitis treatment; weighted average counts at baseline, after treatment, one month, 6 weeks, 3 months, and 6 months; mean absolute counts deviation from the baseline.

	Baseline	After treatment	1 month	6 weeks	3 months	6 months
	1.04 ± 1.30 (20) c.	0.57 ± 0.34 (20) i.	7.18 ± 0.88 (11) e.	2.41 ± 2.44 (9) g.	6.06 ± 2.18 (11) e.	1.06 ± 2.11 (9) g.
	1.27 ± 1.11 (20) d.	1.18 ± 0.23 (20) j.	5.67 ± 2.96 (11) f.	0.39 ± 1.17 (9) g.	5.47 ± 2.91 (11) f.	0.44 ± 1.31 (9) g.
	6.10 ± 2.34 (11) e.			1.39 ± 2.15 (9) g.	0.39 ± 0.58 (20) c.	1.59 ± 2.41 (9) g.
	6.43 ± 2.22 (11) f.			1.35 ± 2.26 (10) h.	0.65 ± 1.19 (20) d.	2.02 ± 2.19 (10) h.
	2.46 ± 1.97 (9) g.			1.81 ± 2.35 (10) h.	1.53 ± 2.39 (9) g.	1.44 ± 2.33 (10) h.
Prevotella intermedia	1.13 ± 1.71 (9) g.			1.73 ± 2.38 (10) h.	0.44 ± 1.32 (9) g.	1.45 ± 2.34 (10) h.
	1.72 ± 2.07 (9) g.				1.00 ± 1.99 (9) g.	
	2.04 ± 2.28 (10) h.				1.40 ± 2.32 (10) h.	
	1.92 ± 2.50 (10) h.				1.42 ± 2.33 (10) h.	
	1.89 ± 2.43 (10) h.				1.49 ± 2.40 (10) h.	
	1.93 (20) i.					
	1.95 (20) j.					
X	1.45 ± 0.10	0.88 ± 0.21	6.42 ± 1.54	1.49 ± 0.88	2.00 ± 0.71	1.35 ± 0.88
D		-0.57 ± 0.23	+4.97 ± 1.54	+0.04 ± 0.89	-0.55 ± 0.61	-0.10 ± 0.78

Abbreviations: weighted average, "X"; mean absolute deviation from the baseline, "*D*"; NSMD plus air-polishing plus diode laser plus aPDT, "c"; NSMD plus air-polishing plus diode laser, "d"; NSMD plus probiotics, "e"; NSMD, "f"; NSMD plus air-polishing plus probiotics, "g"; NSMD plus air-polishing, "h"; SMD plus air-polishing plus diode laser plus aPDT, "i"; SMD plus CHX, "j".

Table S8. *Eikenella corrodens* counts (log CFU/mL) of test and control group, extracted from the studies included in the present systematic review; number of peri-implantitis sites treated (n.) and peri-implantitis treatment; weighted average counts at baseline, after treatment, one month, and 3 months; mean absolute counts deviation from the baseline.

	Baseline	1 month	3 months
Eikenella corrodens	4.36 ± 2.94 (11) e.<br>5.72 ± 1.12 (11) f.	4.48 ± 2.99 (11) e.<br>5.00 ± 1.88 (11) f.	3.77 ± 2.66 (11) e.<br>4.96 ± 1.79 (11) f.
X	5.04 ± 1.57	4.74 ± 1.77	4.37 ± 1.60
D		-0.30 ± 2.37	-0.67 ± 2.24

Abbreviations: weighted average, "X"; mean absolute deviation from the baseline, "*D*"; NSMD plus probiotics, "e"; NSMD, "f".

Table S9. *Aggregatibacter actinomycetemcomitans* counts (log CFU/mL) of test and control group, extracted from the studies included in the present systematic review; number of peri-implantitis sites treated (n.) and peri-implantitis treatment; weighted average counts at baseline, after treatment, one month, 6 weeks, 3 months, and 6 months; mean absolute counts deviation from the baseline.

	Baseline	After treatment	1 month	6 weeks	3 months	6 months
Aggregatibacter actinomycetemcomitans	0.91 ± 0.80 (20) c.<br>1.12 ± 0.86 (20) d.<br>0.00 ± 0.00 (11) e.<br>0.00 ± 0.00 (11) f.<br>3.09 ± 2.54 (9) g.<br>3.56 ± 2.26 (9) g.<br>3.61 ± 2.27 (9) g.<br>3.74 ± 2.47 (10) h.<br>2.76 ± 2.10 (10) h.<br>3.24 ± 1.87 (10) h.<br>1.79 (20) i.<br>1.81 (20) j.	0.45 ± 0.04 (20) i.<br>1.04 ± 0.12 (20) j.	0.00 ± 0.00 (11) e.<br>0.00 ± 0.00 (11) f.	3.71 ± 1.66 (9) g.<br>3.50 ± 2.11 (9) g.<br>3.52 ± 2.71 (9) g.<br>3.67 ± 2.30 (10) h.<br>2.78 ± 2.09 (10) h.<br>2.67 ± 2.45 (10) h.	0.47 ± 0.64 (20) c.<br>0.61 ± 0.62 (20) d.<br>0.00 ± 0.00 (11) e.<br>0.00 ± 0.00 (11) f.<br>3.62 ± 2.43 (9) g.<br>2.80 ± 2.26 (9) g.<br>3.83 ± 1.78 (9) g.<br>3.43 ± 2.33 (10) h.<br>2.53 ± 1.83 (10) h.<br>2.71 ± 2.07 (10) h.	2.44 ± 2.41 (9) g.<br>2.42 ± 2.44 (9) g.<br>3.37 ± 2.19 (9) g.<br>2.45 ± 2.92 (10) h.<br>2.88 ± 2.06 (10) h.<br>2.36 ± 2.14 (10) h.
X	1.45 ± 0.30	0.75 ± 0.06	0.00 ± 0.00	3.29 ± 0.92	1.49 ± 0.47	2.65 ± 0.99
D		-0.70 ± 0.31	-1.45 ± 0.30	+1.84 ± 0.97	+0.04 ± 0.17	+1.20 ± 0.89

Abbreviations: weighted average, "X"; mean absolute deviation from the baseline, "*D*"; NSMD plus air-polishing plus diode laser plus aPDT, "c"; NSMD plus air-polishing plus diode laser, "d"; NSMD plus probiotics, "e"; NSMD, "f"; NSMD plus air-polishing plus probiotics, "g"; NSMD plus air-polishing, "h"; SMD plus air-polishing plus diode laser plus aPDT, "i"; SMD plus CHX, "j".

Table S10. Total anaerobic bacteria counts (log CFU/mL) of test and control group, extracted from the studies included in the present systematic review; number of peri-implantitis sites treated (n.) and peri-implantitis treatment; weighted average counts at baseline, after treatment, 3 months, and 6 months; mean absolute counts deviation from the baseline.

	Baseline	After treatment	3 months	6 months
Total Anaerobic Bacteria	9.23 ± 3.06 (13) k.<br>12.02 ± 1.90 (12) f.<br>2.35 ± 0.02 (20) i.<br>2.37 ± 0.03 (20) j.	0.98 ± 0.20 (20) i.<br>1.58 ± 0.34 (20) j.	9.43 ± 1.85 (13) k.<br>9.05 ± 2.74 (12) f.<br>1.50 (20) i.<br>1.86 (20) j.	8.66 ± 2.55 (13) k.<br>8.80 ± 2.49 (12) f.<br>2.06 (20) j.<br>1.77 (20) i.
X	5.51. ± 0.71	1.28 ± 0.20	4.58 ± 0.63	4.53 ± 0.69
D		-4.23 ± 0.46	-0.93 ± 0.67	-0.98 ± 0.70

Abbreviations: weighted average, “X”; mean absolute deviation from the baseline, “*D*”; NSMD, “f”; SMD plus air-polishing plus diode laser plus aPDT, “i”; SMD plus CHX, “j”; Er:YAG, “k”.

Table S11. Total peri-implant microbial load (log CFU/mL) recorded in the included RCTs of test and control group, extracted from the studies included in the present systematic review; number of peri-implantitis sites treated (n.) and peri-implantitis treatment; weighted average counts at baseline, after treatment, one month, and 3 months; mean absolute counts deviation from the baseline.

	Baseline	1 month	3 months
Total Bacteria	9.05 ± 1.11 (11) e.<br>9.31 ± 0.67 (11) f.	9.46 ± 0.93 (11) e.<br>9.26 ± 0.66 (11) f.	8.96 ± 1.10 (11) e.<br>9.33 ± 0.74 (11) f.
X	9.18 ± 0.65	9.36 ± 0.57	9.14 ± 0.66
D		+0.18 ± 0.86	-0.04 ± 0.93

Abbreviations: weighted average, “X”; mean absolute deviation from the baseline, “*D*”; NSMD plus probiotics, “e”; NSMD, “f”.
