# Supplementary material for: Peri-Implantitis-Associated Microbiota before and after Peri-Implantitis Treatment, the Biofilm “Competitive Balancing” Effect: A Systematic Review of Randomized Controlled Trials
Source: Microorganisms. 2024 Sep 28;12(10):1965. doi: 10.3390/microorganisms12101965 (PMC11509653; doi:10.3390/microorganisms12101965)
Supplement: Supplementary file 1 [file microorganisms-12-01965-s001.zip › Supplementary File S2.pdf]

Peri-Implantitis-Associated Microbiota before and after Peri-Implantitis Treatment, the Biofilm “Competitive Balancing” Effect: A Systematic Review of Randomized Controlled Trials

Federica Di Spirito [1,*], Massimo Pisano [1,†], Maria Pia Di Palo [1,†], Gianluigi Franci [1], Antonio Rupe [1], Antonino Fiorino [2,‡] and Carlo Rengo [1,*,‡]

Supplementary File S2 – Quality Assessment of included studies

3.8 Quality Assessment

The risk of bias and the quality assessment of the RCTs included in the present systematic review were reported in Table S12 and Figure S1.

Table S12. Assessment of the six domains (randomization process, effect of assignment to intervention, effect of adhering to intervention, missing outcome data, measurement of the outcome and selection of the reported studies) for each RCT included in the present systematic review through the RoB 2 tool [36].

	**Randomization process (item 1)**			**Effect of assignment to intervention (item 2.a)**							**Effect of adhering to intervention (item 2.b)**						**Missing outcome data (item 3)**				**Measurement of the outcome (item 4)**					**Selection of the reported studies (item 5)**		
	1.1	1.2	1.3	2.1	2.2	2.3	2.4	2.5	2.6	2.7	2.1	2.2	2.3	2.4	2.5	2.6	3.1	3.2	3.3	3.4	4.1	4.2	4.3	4.4	4.5	5.1	5.2	5.3
Almohareb et al. [37]	Y	PN	N	NI	PY	N	NA	NA	Y	NA	NI	PY	N	N	N	Y	NI	PN	PN	NI	PN	N	N	NA	NA	Y	N	N
Arisan et al. [38]	NI	PN	NI	PY	PY	N	NA	NA	Y	NA	PY	PY	N	N	N	Y	Y	NA	NA	NA	PY	N	PY	NI	N	Y	N	N
Bassetti et al. [39]	NI	PN	Y	NI	N	NA	NA	NA	PN	PN	NI	N	NA	N	N	NA	Y	NA	NA	NA	N	N	N	NA	NA	Y	N	N
Birang et al. [47]	Y	PY	NI	N	N	NA	NA	NA	Y	NA	N	N	NA	N	N	NA	Y	NA	NA	NA	NI	N	NI	NI	N	Y	N	N
Bombeccari et al. [40]	NI	PN	N	PY	PY	N	NA	NA	PY	NA	PY	PY	N	N	N	Y	NI	PN	PN	NI	PY	N	N	NA	NA	Y	N	N
Cha et al. [41]	Y	Y	N	PN	N	NA	NA	NA	PY	NA	PN	N	NA	N	PY	NI	PY	NA	NA	NA	N	N	NI	PN	N	Y	N	N
Chen et al. [42]	Y	Y	N	NI	NI	NA	NA	NA	Y	NA	NI	NI	NA	N	N	NA	Y	NA	NA	NA	NI	N	NI	PN	N	Y	N	N
Galofré et al. [43]	NI	PN	N	N	N	NA	NA	NA	Y	NA	N	N	NA	N	N	NA	PY	NA	NA	NA	NI	N	N	PY	PY	Y	NI	NI
Laleman et al. [44]	Y	Y	N	N	N	NA	NA	NA	Y	NA	N	N	NA	N	N	NA	PY	NA	NA	NA	Y	N	NI	PY	N	Y	N	N
Passariello et al. [45]	NI	Y	N	Y	N	NA	NA	NA	Y	NA	Y	N	NA	N	N	NA	NI	PN	PN	NI	Y	N	NI	PN	N	Y	N	N
Shibli et al. [46]	Y	Y	NI	NI	NI	NA	NA	NA	Y	NA	NI	NI	NA	N	N	NA	NI	PN	PN	NI	NI	N	NI	NI	N	Y	N	N

Abbreviations: Yes, “Y”; Probably Yes, “PY”; Probably No, “PN”; No, “N”; No Information, “NI”; Not applicable, “NA”.

A.

Legend: + Yes (low risk); ? Unclear; − No (High risk)

	Almohareb et al. [37]	Arisan et al. [38]	Bassetti et al. [39]	Birarng et al. [47]	Bombeccari et al. [40]	Cha et al. [41]	Chen et al. [42]	Galofré et al. [43]	Laleman et al. [44]	Passariello et al. [45]	Shibli et al. [46]
Randomization process	−	−	−	+	−	+	+	?	+	+	+
Effect of assignment to intervention	+	+	?	+	+	+	+	+	+	+	+
Effect of adhering to intervention	?	?	+	+	?	−	+	+	+	+	+
Missing outcome data	+	+	+	+	+	+	+	+	+	+	+
Measurement of outcome	+	−	+	?	−	+	?	+	−	−	?
Selection of the reported result	+	+	+	+	+	+	+	?	+	+	+
Quality	−	−	−	?	−	−	?	?	−	−	?

B.

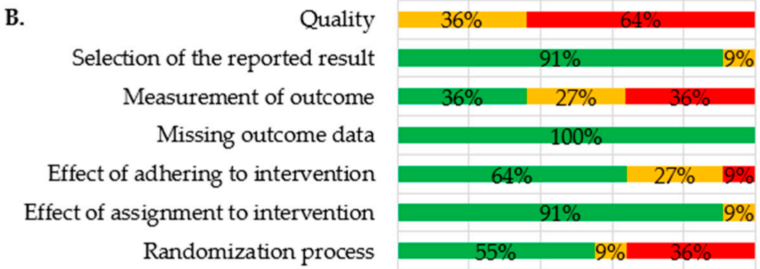

Figure S1. A. Risk of bias judgment of the six domains (randomization process, effect of assignment to intervention, effect of adhering to intervention, missing outcome data, measurement of the outcome and selection of the reported studies) and quality assessment for each study included. **B.** Risk of bias judgment and quality assessment presented as percentage of low risk, unclear and high risk.
